# Nanofiber-based systems intended for diabetes

**DOI:** 10.1186/s12951-021-01065-2

**Published:** 2021-10-12

**Authors:** Hassan Maleki, Kamyar Khoshnevisan, Sayed Mahmoud Sajjadi-Jazi, Hadi Baharifar, Maryam Doostan, Nazanin Khoshnevisan, Farshad Sharifi

**Affiliations:** 1 Nano Drug Delivery Research Center, Health Technology Institute, Kermanshah University of Medical Sciences, Kermanshah, Iran; 2 Medical Nanotechnology and Tissue Engineering Research Center, Shahid Beheshti University of Medical Sciences, Tehran, Iran; 3 Department of Tissue Engineering and Applied Cell Sciences, School of Advanced Technologies in Medicine, Shahid Beheshti University of Medical Sciences, Tehran, Iran; 4 Cell Therapy and Regenerative Medicine Research Center, Endocrinology and Metabolism Molecular-Cellular Sciences Institute, Tehran University of Medical Sciences, 1411713137 Tehran, Iran; 5 Department of Medical Nanotechnology, Applied Biophotonics Research Center, Science and Research Branch, Islamic Azad University, 1477893855 Tehran, Iran; 6 Research and Development Team, Evolution Wound Dressing (EWD) Startup Co., Tehran, Iran; 7 Elderly Health Research Center, Endocrinology and Metabolism Population Sciences Institute, Tehran University of Medical Sciences, 1411713137 Tehran, Iran; 8 Endocrinology and Metabolism Research Center, Endocrinology and Metabolism Clinical Sciences Institute, Tehran University of Medical Sciences, 1411713137 Tehran, Iran

**Keywords:** Nanofiber, Diabetic wound, Electrospinning, Scaffold, Wound dressing, Delivery systems, Cell transplantation

## Abstract

Diabetic mellitus (DM) is the most communal metabolic disease resulting from a defect in insulin secretion, causing hyperglycemia by promoting the progressive destruction of pancreatic β cells. This autoimmune disease causes many severe disorders leading to organ failure, lower extremity amputations, and ultimately death. Modern delivery systems e.g., nanofiber (NF)-based systems fabricated by natural and synthetic or both materials to deliver therapeutics agents and cells, could be the harbinger of a new era to obviate DM complications. Such delivery systems can effectively deliver macromolecules (insulin) and small molecules. Besides, NF scaffolds can provide an ideal microenvironment to cell therapy for pancreatic β cell transplantation and pancreatic tissue engineering. Numerous studies indicated the potential usage of therapeutics/cells-incorporated NF mats to proliferate/regenerate/remodeling the structural and functional properties of diabetic skin ulcers. Thus, we intended to discuss the aforementioned features of the NF system for DM complications in detail.

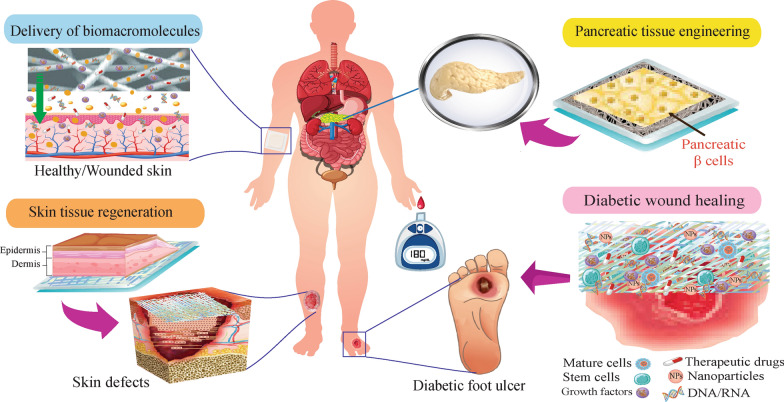

## Introduction

Nowadays, diabetes mellitus (DM) acts as a colossal problem and life-threatening disease for humanity health by globally escalating prevalence. DM is recognized by glucose level dysregulation in blood result from defects in insulin secretion by pancreas (type 1 DM) and/or impair the response of body to insulin (type 2 DM) [1, 2]. In 2019, all over the world, 463 million people suffered from DM, with 4.2 million deaths yearly and it is estimated to strike about 700 million by 2045 [3]. World Health Organization (WHO) expressed that DM will turn into the seventh greatest reason for mortality in 2030 [4]. Numerous macrovascular/microvascular complications are associated with DM as an effect on vital organs in the body, including high risks of heart disease, peripheral vascular disease, kidney failure, neuropathy, retinopathy, and even lower extremity amputations [5]. As a result, exogenous insulin administration and other therapeutics are indispensable for regulating blood glucose levels. The conventional route of insulin delivery may be accompanied by pain, needle phobia, local tissue-damaging, and decreased compliance, as well as the risk of infection [6].

**Fig. 1:**
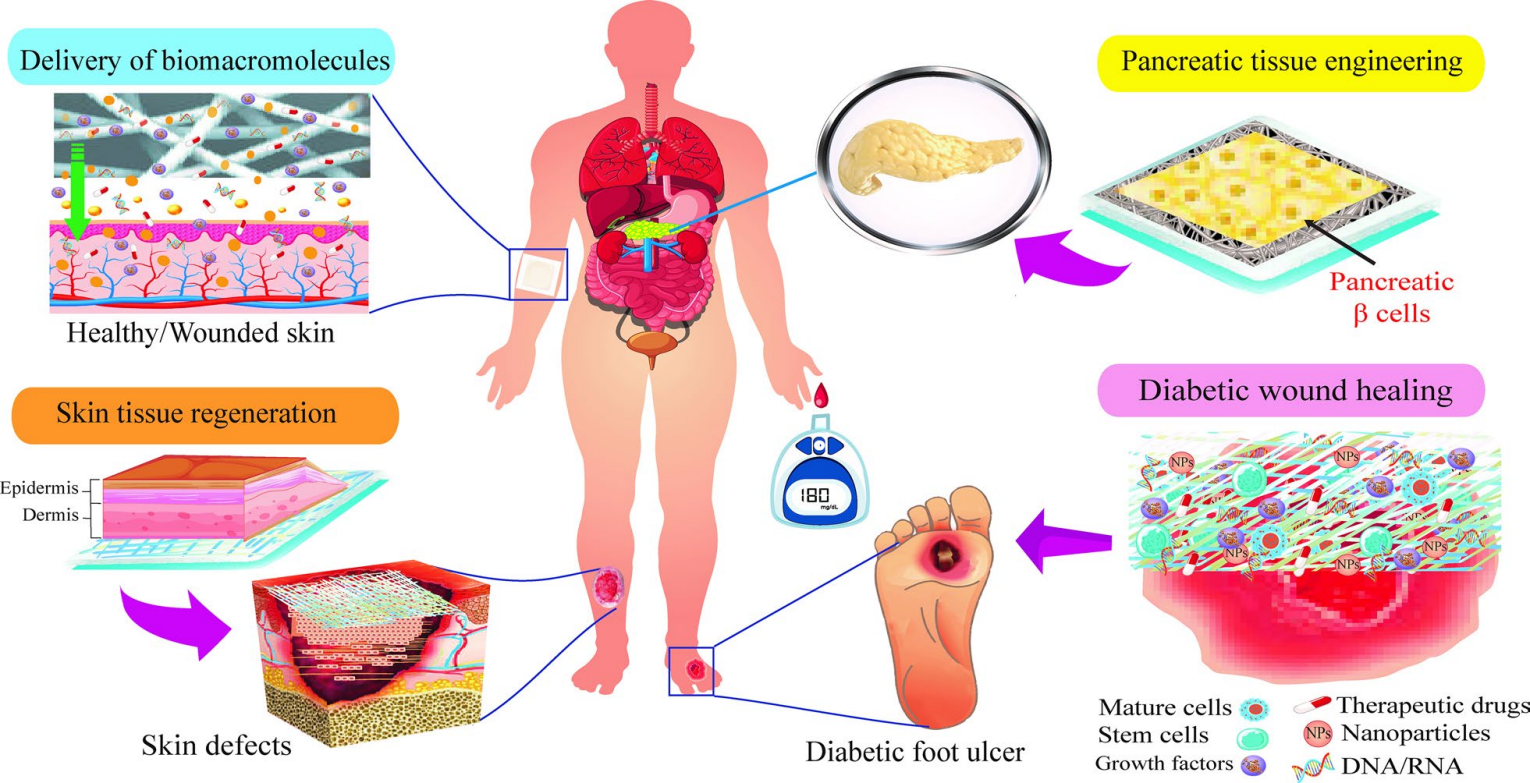
Utilization of nanofiber-based systems for treatment of DM through several approaches

To obviate these restrictions, an immense variety of delivery methods were investigated to control blood glucose levels, including oral, nasal, pulmonary, and transdermal approaches, etc. [7–9]. Nevertheless, each of these methods encounters with some limitations including poor permeability across the barriers of body, possible allergic or irritation reactions, difficulty to achieve high plasma drug concentration, and low or variable bioavailability owning to degradation by proteolytic enzymes [10–17]. Therefore, using alternative delivery strategies is imperative to prevent limitation/problems and improve effectiveness as well satisfaction of diabetic patients. A number of nanostructure-based delivery systems were studied to conquer different DM-associated complications [18–20]. Therefore, NF-based systems have presented tremendous capabilities as delivery systems and as artificial scaffolds to deliver therapeutics agents and cells (Fig. 1).

**Fig. 2:**
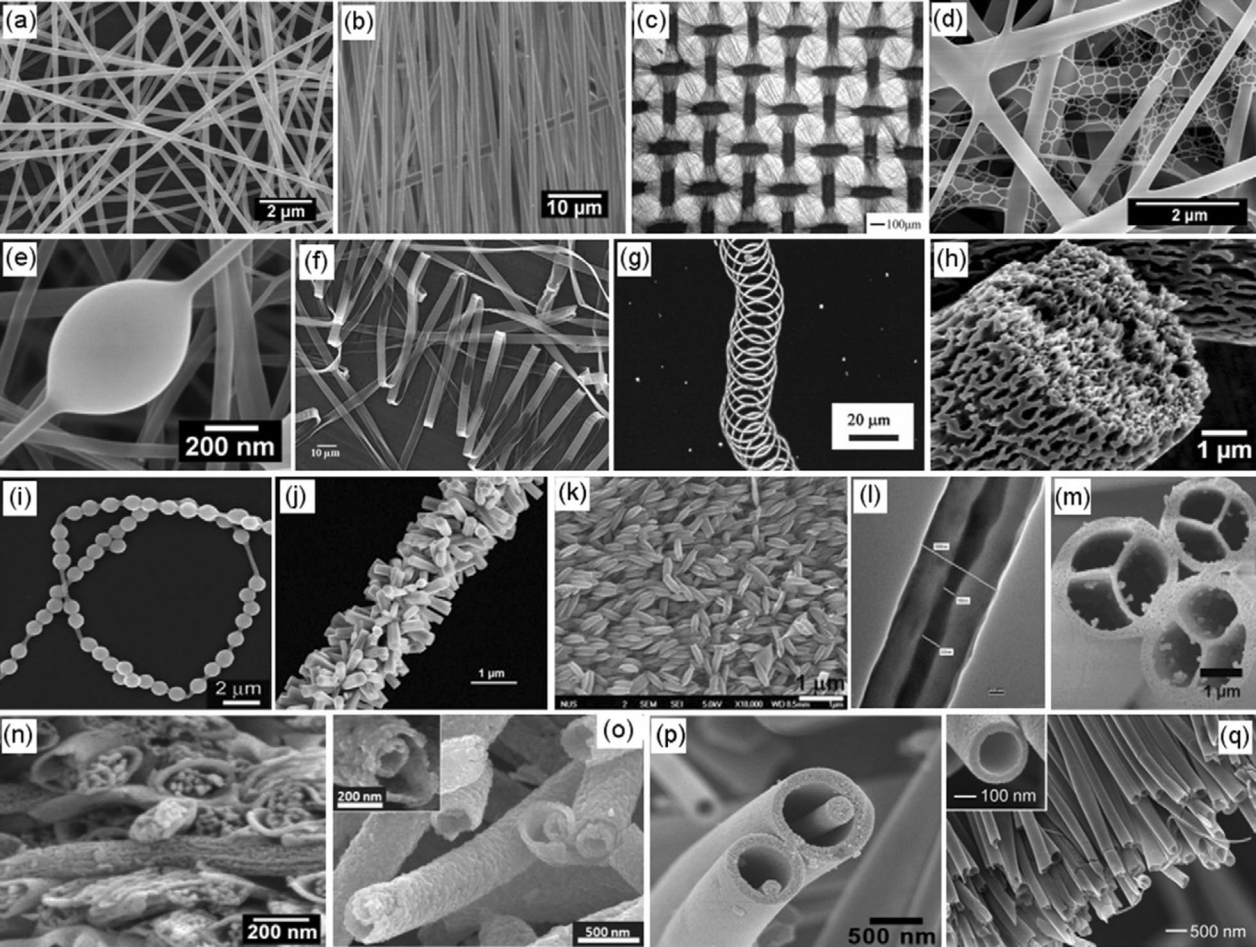
Fibers with multifarious morphologies prepared by electrospinning. **a**–**d** Different NF assembly morphologies: **a** random oriented, **b** aligned as well as (**c**) patterned and (**d**) spider-web-like nano-fiber/net structures. **e**–**q** Various single NFs with (**e**) bead-on-string, (**f**) ribbon-like, (**g**) helical, (**h**) porous [30], (**i**) necklace-like, (**j**) firecracker-shaped, (**k**) rice grain-shaped, (**l**) core—shell, (**m**) multichannel tubular, (**n**) multi-core cable-like, (**o**) tube-in-tube, (**p**) nanowire-in-microtube and (**q**) hollow structures. Reproduced with permission from Ref. [21]

As a delivery system or reservoirs, NFs can provide an adequate matrix for encapsulation and incorporation of therapeutic agents as well as able to prevent destruction before reaching their target sites with high-efficiency, and low-adverse effects. Such structures possess high flexibility in producing various morphologies (Fig. 2) [21], high drug-loading capacity (up to 60%), and encapsulation efficiency (up to 100%), as well as have the potential to deliver their content [22, 23]. Therapeutic agents are loaded in the fibers by different methods, including a combination of agent with the polymer solution before spinning, producing core/shell structures through coaxial spinning, attaching active agents on the surface of the fiber, post-fabrication surface modification, and grafting on the surface [24, 25]. These methods can be applied for more precise control over release kinetics and achieve timely release of therapeutic agents.

Artificial scaffolds can create three-dimensional (3D) fibrous frameworks that mimic the natural extracellular matrix (ECM) multi-fibril networks in design and structure which are mostly used as ECM substitutes to support the vital functions of cells [26, 27]. NF scaffolds with architectural similarity to native ECM can provide an immense surface area for cell-scaffold interaction/adherence and effective exchange for oxygen and nutrition transportation. NFs can be incorporated with ECM proteins, growth factors (GF), and nanomaterials to promote the formation of tissue-like structures for tissue-engineered implantation/transplantation [28].

Multifarious natural materials and synthetic polymers were exploited to synthesis NF structures for DM treatment. In general, natural polymers showed superior biocompatibility, suitable biodegradation, and significantly lower immunogenicity, whereas synthetic polymers can form electrospun much easier with good mechanical strength and high flexibility. To take the maximum advantages from those materials, using a combination strategy is recommended.

NF–based systems regarding several approaches were broadly applied in recent years for DM treatment (Fig. 1). Delivery of biomacromolecules e.g., insulin, GFs, small interfering RNA, as well as anti-diabetic chemical agents, is one of the most prominent features of NF–based structures for DM treatment [29, 30]. Insulin can be incorporated/coated in NF patches to be administered via dermal/transdermal or by other routes e.g., sublingual to decline plasma glucose level. In particular, insulin-loaded dressings can promote the formation of a wound matrix and accelerate wound healing in patients with DM [31, 32]. Furthermore, Genes and GFs can be incorporated within or onto NFs [33, 34]. These biomolecules stimulate cell proliferation, differentiation, angiogenesis, tissue repair, and regeneration. Hence, using locally controlled and efficient delivery to target cells e.g. NF scaffolds can achieve to further increase delivery efficiency or extend function duration, thereby could be fruitful to induce the healing of diabetic ulcers and promote cell’s activities to skin or pancreatic tissue engineering (TE).

Diabetic Wound (DW) remains a major clinical challenge due to impaired healing process composed of multifactorial which resulted from peripheral neuropathy, impaired vascular function, impaired angiogenesis, and/or chronic inflammation as well as microbial infection in chronic wounds [35, 36]. By the inhibition of wound healing process, these complications resulted in delayed healing or even non-healing so which caused to 15% surgical amputation of all diabetic patients, despite a carefully calculated diet and intensive medical treatment [37]. Consequently, desirable wound dressings with biomimetic multifunctional features are indispensable to provide hemostasis, moisture retention, antibacterial effect, regeneration promotion capability, and ability to deliver bioactive agents. NF dressings/scaffolds are emerging technologies in wound healing making ECM-like networks that can deliver herbal/chemical drugs, GFs, and nanomaterials in a controlled manner as well as propel and promote cell proliferation and differentiation [28, 38, 39].

Pancreatic TE and β cell replacement are another emerging areas in which NFs serve as a ECM-mimicking matrix for support and growth of islet β‐cells and differentiation of stem cell-generated β cells to treat DM. Using a bio-inspired hybrid scaffold is a novel approach to simulate pancreatic micro/nanoenvironment for preserving survival and function of cells as well as promoting cell differentiation into insulin-producing cells (IPCs) [27, 40]. It seems that these 3D scaffolds are a considerable candidate to hinder the limitations of current β cell production and islet transplantation (IT) to use in clinical pancreatic TE application.

Based on above-mentioned knowledge, the present study is focused on the capability of NFs-based platforms for therapeutics delivery, wound healing, and TE for DM treatment. At first, a brief description of electrospinning (ES) method as the most applicable technique for the fabrication of NFs and the characteristics of natural and synthetic polymers applied for NF preparation are described. Then, we have reviewed various studies related to the incorporation of drugs/genes/GFs in NFs-based delivery systems. Afterward, the capability of NFs for DW healing/dressing is discussed. Finally, we addressed the usability of NF scaffolds to function as artificial ECM in pancreatic β cells replacement and TE for DM.

### Fabrication methods and characteristics of NFs

As a significant matrix/scaffold, NFs are featured with small diameter, high porosity, high specific surface area, controlling of their composition, tailoring mechanical and surface features, and ease of synthesis [27, 41]. These structures possess proper sponginess for the absorption of exudates, highly permeable to water vapor, allowed an effective exchange of oxygen, water, and nutrient, and also can be functionalized with different molecular moieties [38]. The common strategies to create NFs include drawing solution blowing, self-assembly, template synthesis, phase separation, and ES [42, 43]. ES techniques are considered the most used technique to enable the fabrication of continuous fibers in the nanoscale dimension from a wide-ranging of either natural and synthetic polymer or a combination of both polymers. ES acts as a remarkably robust, versatile, and one-step technique for fabricating ordered and complex NF architectures using a high voltage electrical field applied to a polymer solution or melt [44, 45]. Furthermore, fibers with varied morphologies could be fabricated via the control of processing condition and modifying standard set up of ES to produce nonwoven fibers with randomly aligned, straight aligned, core–shell, ribbon, porous structures, and so on (Fig. 2) [21, 46].

Electrospun NFs with desirable physical characteristics and high uniformity structure can be obtained by modulating the effective parameters, including parameters related to polymer solution, the electrospinning equipment, and environmental condition. Amid these, polymer solution parameters have a critical role in the formation of NFs with a broad range of sizes and morphologies, including concentration and molecular weight of polymer, solution conductivity, and solvent volatility. Viscosity and surface tension of polymer solution possess a decisive role in the morphology and size of electrospun NFs and are directly under the influence‏ of the molecular weight of polymer and solution concentration. Generally, low solution concentration that caused to low viscosity and high surface tension of the solution leads to the formation of beads and droplets, while very high solution concentration leads to blocking the capillary tip and disturbing rate of charged polymer flow leading to appear helix-shaped fibers and/or fibers could not be formed [47, 48]. Besides, the molecular weight of polymer in a range suitable is necessary for the entanglement of polymer chains in solutions so that low molecular weight solutions resulted in the formation of beads instead of fibers and high molecular weight inclined to form microribbons [47, 49]. A proper solvent is crucial for the dissolution of polymer and the formation of fibers during the electrospinning jet elongation through the evaporation of solvent and phase separation. Applying solvents with a higher evaporation rate and boiling point can lead to the generation of surface roughness and pores on the surface fiber [48, 50]. The other important parameter, the solution conductivity is determined by types of polymer, solvent, and salt. In this regard, by increasing conductivity the electrospinning jet carries more charges as well as NFs with smaller diameters and fewer beads can be produced [49, 50]. Furthermore, the fabrication of NFs is affected by parameters related to the electrospinning process (e.g., applied voltage, tip-to-collector distance, and feed rate) and environmental parameters in the spinning chamber (e.g., temperature, humidity, and air velocity), which all of these parameters must be optimized [47–50].

In terms of length of NFs, they are produced and elongated continuously from a few ‏µ‏m to tens of meters with distinctive orientation and alignment that are required to cater to particular demands in the biomedical field [51, 52]. Moreover, diameter of NFs is proportional to the various fabrication parameters as mentioned earlier and could be adjusted from nanometer to microns [52]. Such extraordinarily small diameters can provide an extremely high surface-to-mass ratio (ranging from 1 to 35 m/g depending on the fibers’ diameter), high and interconnected porosity as well as high accessible sites for functionalization and immobilization [53]. Besides, with control over the NF diameter, its mode of encapsulation, and varying the morphology to core–shell type, the release kinetic from NFs mats can be modulated [54]. The diameter and morphology of electrospun NFs possess similar to the human ECM in terms of scale and morphology, thereby they have ability to accelerate the process of cell functions e.g., adhesion, proliferation, and differentiation [54, 55].

The mechanical properties of nanofibrous scaffolds/mats are depended on the different structural parameters such as fiber diameter, alignment, porosity, and spatial distribution of NFs [43]. Both elastic modulus and strength of nanofibers significantly increase with declining fiber diameter that is attributed to increment in the crystallinity, the densely packed lamellae, and aligned fibrillar structures [56].

Porosity is another outstanding parameter of NFs that could be controlled the pore size distribution, by forming a highly open porous architecture and interconnected pore structure [57, 58]. Thus, they have a beneficial effect on cell survival and proliferation as well as permit the transport of fluids and gases, the diffusion of nutrients, and prevention from bacterial infections [48]. Meanwhile, conventional hydrogels as another 3D cross-linked polymer matrices are capable of imbibing high water content, swelling without dissolving, and providing high porosity and elasticity [59, 60]. However, such hydrogels often lack fibrous structures and the anisotropy features of native tissue ECM as well as possess insufficient mechanical strength [61]. Besides, precise control over porosity and the microarchitectural features of hydrogels still remained challenging issues [62]. In addition, several electrospun materials can able to form hydrogel NF systems with combined the desirable properties of both NF and hydrogel [63]. Particularly, porosity and swelling behavior significantly increased in hydrogel NFs due to their small pore size compare with conventional hydrogels [63].

### Characteristics of natural materials to NFs preparation

Since the components of designed NF scaffolds should be located in proximity to native ECM, thereby they are frequently fabricated of natural, biodegradable, and biocompatible materials. Naturally occurring materials have garnered much interest in the field of biomedical applications due to better biocompatibility, biodegradability, low immunogenicity, and moderate mechanical stability compared to synthetic polymers [64]. Various biopolymers were utilized to make NFs for DM treatment which can be categorized into two major groups, polysaccharides and polypeptides; for instance, collagen, gelatin (Gel), silk fibroin (SF) of polypeptides, cellulose, chitosan (CS), hyaluronic acid (HA), and alginate from polysaccharides.

Collagen is the most prevalent fibrous protein in the ECM of connective tissues and comprised up to 30% of the total protein mass of a multicellular animal [65]. Collagen is formed by self-assembly of collagen triple helices, providing tensile strength, regulate cell adhesion, support chemotaxis and migration, and direct tissue development [66]. Denatured collagen, or Gel, has attracted a great deal of interest in NF synthesis, owning to its biological origin, biocompatibility, and excellent biodegradability with low immunogenicity and commercial availability at low cost [67]. The gel is derived from partial physical or chemical hydrolysis of collagen which is a soluble and amphoteric protein, enabling it to form a thermally reversible network in water because of alkaline and acidic amino acid residues [68, 69].

SF is amongst the most impressive natural materials is that abundantly utilized in a multitude of biomedical applications. SF is the main component of silk produced by some creatures like silkworms (*Bombyx mori*) [44]. The raw silk consists of two parallel fibroin filaments wrapped with the glue-like sericin protein [70]. SF is an amphiphilic block copolymer made up of hydrophobic and hydrophilic segments endowed with an amalgamation of remarkable tensile strength and toughness, good biocompatibility, perfect proteolytic degradability, and thermostability [44, 70].

Like other groups of biomaterials, polysaccharides are frequently applied to develop NF structures in terms of their innate physicochemical properties. Cellulose is considered the most profuse polysaccharide due to its ubiquitous nature and abundance as well as can be derivate to proper polymers for various utilizations. Thus, the most important acetate ester of cellulose, cellulose acetate (CA), is a biocompatible, biodegradable, renewable compound with excellent chemical resistance [71–73]. CA was broadly used for a broad spectrum of utilities in different engineered architectures/forms e.g., NF structures [74]. Besides, CA is used more than cellulose to make NFs because of more capability to ES to generate nanoscale fibers [71].

As de-acetylated derivative of chitin, CS is a heteropolysaccharide composed of glucosamine and Nacetyl glucosamine units linked by β(1–4) glycosidic bonds. CS showed numerous inherent attributes e.g., biocompatibility, biodegradability, mucoadhesive ability, and anti-bacteria activity [19, 55, 75].

HA is a non-sulfated glycosaminoglycan with the pleiotropic function which is found abundantly through body. HA merits attention for biomedicine applications due to its fascinating properties, comprising biocompatibility, biodegradability, non-immunogenicity, high water retention ability, and high viscoelasticity [28, 69]. Moreover, HA enables to facilitate all phases of wound healing cascade and influences cell activities [19]. HA-based NFs were reported as being very considerable biomaterial for DW healing and developing bio-mimicking scaffolds for pancreatic TE applications.

### Characteristics of synthetic materials to NFs preparation

Synthetic polymers possess especially benefits relative to natural polymers including easier ES with excellent mechanical strength, desired degradation rate, and proper thermal stability [55]. The most profuse synthetic polymers used the fabrication of NFs for diabetics’ treatment can include polycaprolactone (PCL), poly vinyl alcohol (PVA), polylactic acid (PLA), poly lactic-co-glycolide (PLGA) copolymers, polyethylene glycol (PEG) as well as other less commonly used polymers e.g., polyethersulfone (PES), poly 3-hydroxybutyrate-*co*-3-hydroxyvalerate (PHBV), polyacrylonitrile (PAN) and so on. Although these polymers can be separately used to produce NFs, they are mostly applied in combination with other natural and synthetic materials to achieve higher or combined advantages.

PCL is a sufficiently biocompatible and slowly biodegradable polyester with high mechanical strength and good thermal stability which was intensively studied as worthy material for fabrication of 3D architectures [27, 29, 44]. PCL NFs provide mimic natural ECM for TE utilizations which are employed as long-term drug delivery carriers, although its hydrophobic nature resulted in poor cell attachment and proliferation [34, 55]. Combination with other hydrophilic polymers such as collagen, Gel, or CS and also high porosity on the surface of PCL NFs can obviate this limitation. PVA is other widely used synthetic polymers for NF fabrication for treating DM which is a water-soluble, non-toxic, biodegradable, and biocompatible polymer [55]. PVA NFs showed excellent mechanical properties and chemical resistance with a high swelling capacity [19, 55].

PLA is a natural-derived thermoplastic polyester which is very popular for NF fabrication. It is in terms of the unique characteristics such as favorable biocompatibility, excellent bioresorbable, and good solubility [76]. PLGA, the most well-known copolymer, is a food and drug administration (FDA)-cleared synthetic polymer which is widely applied thanks to its unique features e.g., non-toxicity, biocompatibility, and processability [76, 77].

As alluded to above, many types of NFs were applied for DWs treatment; however, unmet need for comprehensive investigation of therapeutic agents and cells delivery via nanofiber-based systems proposed for diabetic mellitus still remained. Thus, we aimed to focus the summary points of nanofiber-based systems effects on DM wounds as a novel therapeutic and preventive approach.

### Insulin delivery

Insulin is a 5.8 kDa protein which plays an important role in regulating metabolism and enhancement of cell growth [78, 79]. The oral use of insulin is still challenging owing to easy degradation in the gastro-intestinal tract. Toward to end of prescribing insulin, several studies were carried out to develop the oral form of this protein [80, 81]. Some studies tried to encapsulate the insulin into the nanoparticle or fibers to prevent the degradation in the stomach and early intestine [82–84]. Besides, insulin can be applied to the skin directly for a sustainable release system. Therefore, we tried to focus on studies considered nanoparticles in adjuvant to insulin in scaffolds to utilize in cell cultures and develop oral insulin.

Asako Nishimura et al. applied insulin as a peptide model (PuraMatrix™, PM), promoting drug delivery after injection of insulin via a self-assembling nanofiber hydrogel scaffold which is called PM-insulin sol (PM-Isol). The findings displayed that the plasma level of insulin was increased with an increment of PM-Isol concentration. Moreover, the bioavailability and hypoglycemic efficiency of insulin was increased after subcutaneous injection of the PM-Isol [37]. Another study by Yan et al. reported that they could construct a film from poly (vinyl alcohol-*co*-ethylene)/CS nanocomposite via a green and facile electrodeposition method. By decreasing pH, the permeability of nanocomposite progressively increased. The releasing behaviors of this component can manage by the external imposing of electric signal [85]. Adnan Haider et al. carried out a study to develop tissue engineering in orthopedic surgery. They revealed that PLGA insulin-grafted hydroxyapatite nanorods composite nanofiber scaffold had increased osteoblastic cell growth. It may indicate that this scaffold released appropriate insulin molecules and insulin to enhance osteoblastic proliferation [86].

**Fig. 3:**
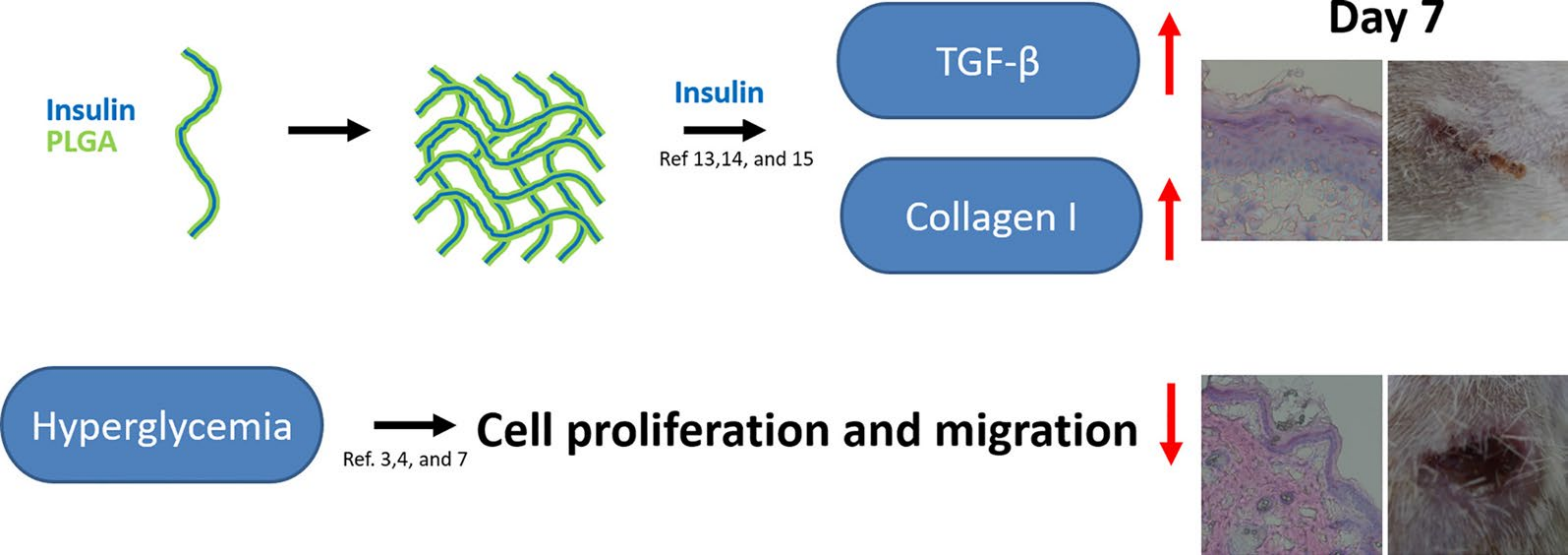
Functionally active insulin released from insulin-loaded nanofibrous scaffolds to accelerate the healing wound Reproduced with permission from Ref. [87]

Shih-Jung Liu et al. developed loaded insulin into biodegradable core–shell nanofibers from PLGA by leading the solution from Insulin and PLGA into two capillary tubes using two pumps. This study revealed that nanofibrous core–shell insulin-loaded scaffold could decrease the quantity of type I collagen and increase the transforming growth factor-beta (Fig. 3) [87].

Other study in Denmark that Karen Stephansen et al. performed applied the bioactive electrospun fish sarcoplasmic protein (FSP) for careering of small protein such as insulin to small intestine cells. Encapsulation of insulin can prevent insulin degradation by chymotrypsin and 12% increase of insulin transportation into cells by the interaction between nanofibers and Caco-2 cells which leads to open of tight junction proteins [88]. In a similar study by S. R. Dhakate tried to develop a transdermal patch from nanofibers of PVA and sodium alginate electrospun composite with loaded insulin. Insulin was released sustainably from the developed nanofiber patch which was compatible with the commercial formulation. An encapsulation efficiency provides a satisfactory indication which obtained nanofibers act as a perfect carrier for sublingually delivery of insulin [89].

Michael G. Lancina et al. used CS electrospun nanofiber polyethylene oxide scaffold to develop a carrier for insulin. Different ratios of Poly (ethylene oxide) (PEO) were utilized to regulate the morphology and physical characteristics of scaffold. They observed that a higher CS: PEO ratio in smaller fibers can result in more rapid insulin release. One interesting finding was that CS: PEO20 fibers 16 times higher preheatable to buccal cells compared to free insulin. They suggested that electrospun CS nanofibers may able to use to produce oral insulin components [90]. Table 1 shows the characteristics of mentioned investigations in detail.

**Table 1:** Various characteristics of nanofibrous delivery systems incorporated with insulin

Type of polymer/material	Diameter of nanofiber (nm)	Applied cell type/animal	Main finding	Refs.
PuraMatrix™	–^a^	Male Wistar rats	PGLmarkedly decreased and maintained up to 24 h via subcutaneous route	[37]
PVA-*co*-PE/CS	100–600	–	Nanofibers with the electrochemically controlled release system	[85]
PLGA/nHA-I	520	Osteoblastic cells (MC3T3-E1)	Accelerate the cell adhesion, proliferation, and differentiation of the osteoblastic cells	[86]
PLGA	432 ± 106	Atrial fibroblasts/prague–Dawley rats	Supported accelerated wound healing and favored epithelial cell proliferation	[87]
FSP	360 ± 37	Caco-2 cells	Physically protect the degradation of insulin and increased transport crossing the cell monolayer	[88]
PVA/NaAlg	300–400	Induced diabetes Wistar rats	The composite nanofibers serve as an ideal carrier for the delivery of insulin via the sublingual route	[89]
CS/PEO	200–2000	3T3-L1 preadipocyte cells/ex-vivo porcine buccal mucosa	Nanofiber mats capable of delivering insulin via the buccal mucosa	[90]

^a^Not available data in the article

*PuraMatrix™* acetyl-(Arg-Ala-Asp-Ala)_4_-CONH_2_, *PGL* plasma glucose level, *PVA* poly (vinyl alcohol), *PE* poly(ethylene), *CS* Chitosan, *PLGA/nHA-I *poly(lactide-*co*-glycolide)/insulin-grafted hydroxyapatite nanorods, *FSP* fish sarcoplasmic protein, *NaAlg* sodium alginate, *PEO* poly(ethylene oxide)

It is believed that the conservation of the bioactive form of several biomolecules like proteins, growth factors, some other hormones, vitamins, and steroids for sustainable release from scaffold was required in tissue engineering. Besides, insulin-loaded scaffolds can be served as a sustainable release form to increase insulin proliferation. Several studies indicated that electrospun scaffolds can be suitable to apply in the wound and a combination of six biomolecules (vitamin C, hydrocortisone, insulin, triiodothyronine, epidermal growth factor, and dihydroxyvitamin D3 needs to add to scaffolds which gradually deliver these components to wound.

### Growth factors and gene therapy

NFs decorates with different GFs and DNA, or RNA molecules are significant tools for diabetic ulcer treatment. Most small molecules delivered using NFs are intended to increase damaged cell viability, promote migration and their proliferation. Furthermore, GFs are used to increase angiogenesis around ulcers which affects healing process (Table 2).

**Table 2:** NFs mediated GFs and genes delivery systems intended for diabetic complications

Type of polymer/material	Incorporated/modified agents	Diameter of nanofiber (nm)	Applied cell type/animal	Main finding	Refs.
HMPA	–^a^	10−20	Male Sprague−Dawley rats	Increase wound recovery, formation, and blood vessel density	[91]
HBPA	VEGF and FGF-2	–	–	Transplant recipients achieved normoglycemia at a higher rate (78%) than control animals	[92]
Peptide/heparin hybrid	HGF	–	Rat insulinoma β-cell line INS-1/adult male rats	HGF-loaded KLD2R/Hep gel improve β-cell survival and insulin secretion	[93]
PCL	GO and VEGF	10,000	HUVECs	Increase expression of the eNOS gene in the VEGF signaling pathway	[93]
PCL-PEG block copolymers	DNA	–	NIH3T3 cells/mice	More effective than naked DNA in terms of in vivo transfection	[94]
PCL–PEG block copolymer	Small interfering RNA	–	Dermal fibroblasts/female C57BL/6 mice	The delivery system increased the MMP-2 gene-silencing and neo-collagen accumulation at the wound sites	[94]
PCL–PEG diblock copolymer	hEGF	–	HDFs/female C57BL/6 mice	High hEGF expression level, significantly accelerated wound recovery rates at diabetic ulcer site	[95]
PCL and PEG	bFGF and EGF	–	HDF cells/female C57BL/7 mice	Release system increases tissue recovery	[96]
PCL/PEG/PCL triblock copolymer	hEGF	–	Human primary keratinocytes/female C57BL/6 mice	The NFs improved in vivo wound healing	[97]
PLGA	Vancomycin, gentamicin, and PDGF	371 ± 162 to 655 ± 206	Human fibroblasts/Sprague–Dawley rats	The NFs increased amount of angiogenesis marker (CD31) and accelerated healing in the early stage	[98]
PLAGA	FTY720	–	C57b16/j male mice	The significantly increased the length density of vessels in the moderately diabetic mice	[25]
PLA-PVA	CTGF	2600 ± 1400	3T3 fibroblasts, HaCat keratinocytes, and EA.hy926 endothelial cells	CTGF loaded core–shell NFs improved cell viability, cell proliferation, and cell migration at ulcer site	[23]
PELA	bFGF	783 ± 129	MEF/skin regeneration for diabetic rats with dorsal wounds	The release system improved collagen deposition and ECM remodeling at diabetic ulcer site	[99]
PLGA/CNC composite	Neurotensin	380 ± 28	Round 0.6-cm-diameter full-thickness dermal wounds in mice	The composite NFs promote rapid healing than control groups during 2 week	[100]
Col/HA	VEGF, PDGF, bFGF and EGF	HA: 486 + 151 Col: 534 ± 128	HUVECs/induced diabetic rats	The delivery system accelerated wound closure rate, with elevated collagen deposition and enhanced maturation of vessels	[101]
Eudragit RL/RS 100	Gentamicin and rhEGF	–	Female C57BL/6 mice	The NFs mesh showed acceptable antibacterial activity and In vivo work induced faster wound healing in dorsal wounds	[102]
PHBV/gelatin methacryloyl	EGF	900 ± 600 to 3500 ± 1800	3T3 fibroblasts, HaCat keratinocytes and EA.hy926 endothelial cells	Promoting keratinocytes, fibroblasts and endothelial cells migration and proliferation and enhanced angiogenesis and in vivo wound healing	[103]

^a^Not available data in the article

*HMPA* Heparin mimetic peptide amphiphile, *HGF* hepatocyte growth factor, *PCL* polycaprolactone, *GO* graphene oxide, *VEGF* vascular endothelial growth factor, *HUVEC* human umbilical vein endothelial cell, *PEG* polyethylene glycol, *hEGF* human epidermal growth factor, *HDF* human dermal fibroblasts, *bFGF* basic fibroblast growth factor, *PLGA* poly lactic-co-glycolide, *PDGF* platelet-derived growth factor, *PLA* polylactic acid, *PVA* poly vinyl alcohol, *CTGF* connective tissue growth factor, *NF* nanofiber, *PELA* poly(ethylene glycol)-poly(dl-lactide), *CNC* cellulose nanocrystal, *MEF* mouse embryo fibroblasts, *HA* hyaluronic acid, *Col* collagen, *PHBV* poly 3-hydroxybutyrate-co-3-hydroxyvalerate

GFs and genes delivery systems via NFs networks could be carried out via two different approaches. In first approach, bioactive molecules (i.e., proteins) form NFs network, which could carry GFs or act alone at the ulcer site. The latter was applied as biocompatible and biodegradable polymers to form NFs networks which act as a carrier.

The heparin-mimetic peptide was used to prepare bioactive NFs networks for IT. NFs were prepared during the self-assembly process, which is driven by noncovalent interactions [91]. Heparin-binding peptide amphiphiles (HBPAs) formed NFs networks via self-assembly process using PLA matrices. Then, the fiber was decorated by vascular endothelial growth factor (VEGF) and fibroblast growth factor 2 (FGF2) for IT. HBPAs protect GFs from proteolysis and activate them for signaling pathway. Moreover, the peptide affected GFs release in vivo and resulted in having more control on release profile [92].

PCL NF was used to design a carrier system for an endothelial growth factor (EGF) and graphene oxide (GO) simultaneously [93]. EGF in mixture with PCL and GO affected nitric oxide synthase 3 genes expression in the vascular VEGF pathway.

The combination of different polymers for NF synthesis was mostly employed in numerous studies due to designing a wide range of NF mesh in terms of physicochemical and mechanical properties. In combination with PEG as diblock copolymer, PCL was used for DNA delivery [94], small interfering RNA [94], plasmid human epidermal growth factor (phEGF) [95], and multiple GFs (i.e., basic fibroblast growth factor (bFGF)/EGF) [96]. Release control of DNA and RNA is usually done using linear polyethyleneimine (LPEI) immobilized on NF. LPEI linker was cleavaged by matrix metalloproteinase existed in high concentration at diabetic ulcer. The release profile of LPEI mediated NFs delivery systems were controlled by LPEI/NFs ratio [94, 95]. Different proteins could be simultaneously immobilized via different physical and chemical methods on NFs. The bFGF was loaded in coaxial electrospun PCL/PEG NFs, then EGF was attached on fiber surface using a simple peptide bond (i.e., amine group of the fiber and carboxyl group of the GF). The capability of NFs to design binary release systems resulted in accelerating wound healing [96]. PCL was electrospun with PCL-PEG block copolymer to form NFs mat having functional amine group on the surface. Then, amine group was used for EGF immobilization. Functional group density affected loading capacity and release profile of EGF, which was simply adjusted by PCL/PCL-PEG ratio [97].

PLA as another common polymer was used as PLGA [25, 98], PVA/PLA [23], poly (ethylene glycol)-poly(dl-lactide) (PELA) [99] for NFs preparation which delivers platelet-derived growth factor (PDGF), Connective tissue growth factor (CTGF), and basic fibroblast growth factor (bFGF), respectively. The electrospun PDGF and PLGA/antibiotic were obtained using different needles to prepare co-axial sheath-core NFs. NFs sustain release antibiotic and GF preventing ulcer infection and promoting cell viability simultaneously [98]. PLGA-decorated cellulose nanocrystal (CNC) was used for the delivery of inflammatory mediators which affected diabetic foot ulcer (DFU) healing. PLGA/CNC NFs were prepared in one step by adding CNC in PLGA solution before ES process. CNC improved PLGA mechanical properties and increased cell response (attachment, migration, and proliferation) at diabetic ulcer sites in vivo [100]. In another work, PVA was used as a core polymer for CTGF delivery in core-sheath NFs. PLA as a sheet resulted in having a porous medium which facilitates release profile control [23].

**Fig. 4:**
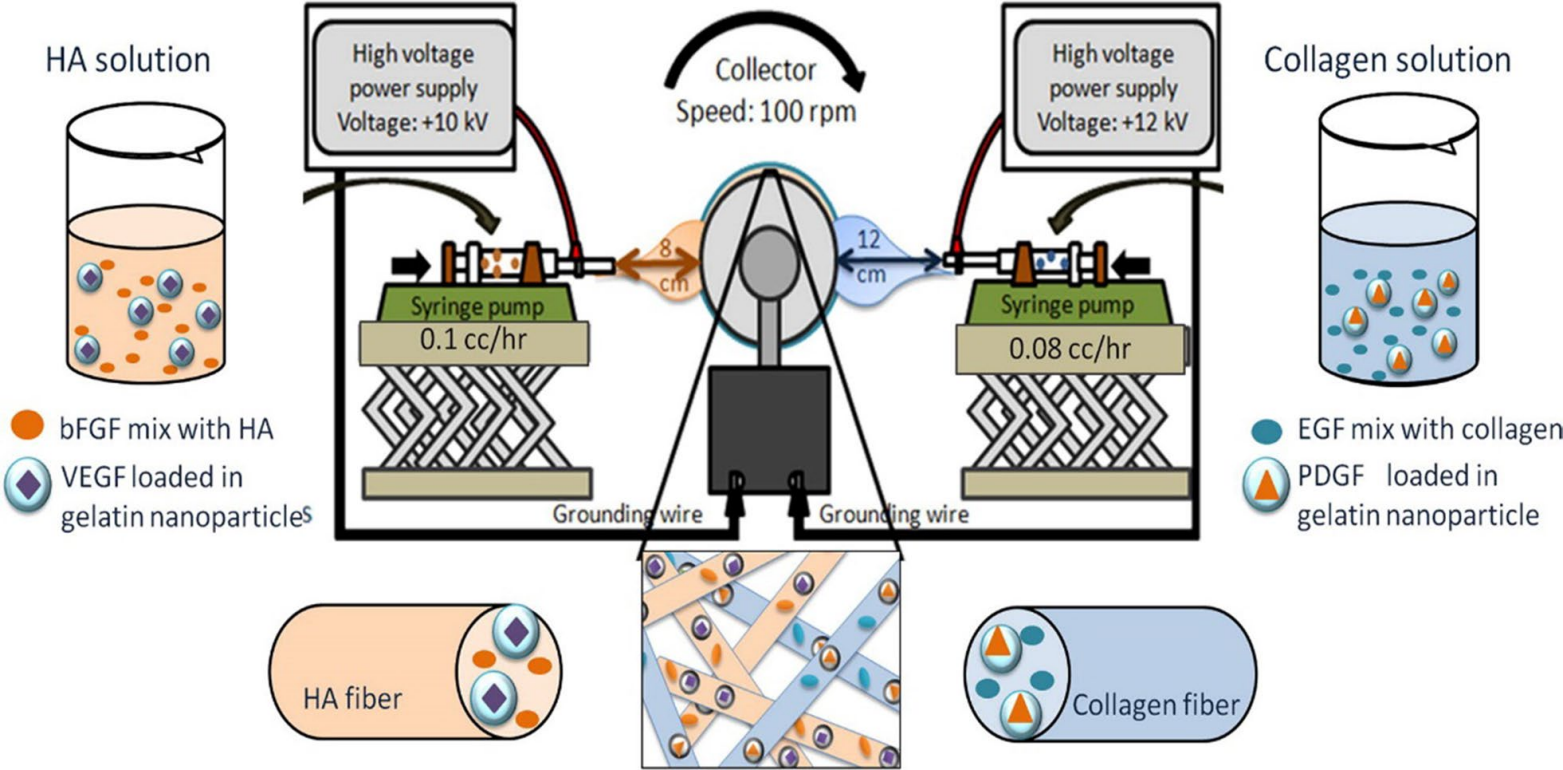
NFs preparation process for immobilization of four different GF using gelation NPs and dual source dual power ES. Reproduced with the permission from reference [101]

NFs can deliver different small molecules to ulcer sites. As mentioned earlier, different proteins and nucleic acids were loaded inside of NFs or attached on the surface of fiber using functional groups. Using nanoparticles (NPs) that carry special GF incorporation by NFs results in increasing release time and loading capacity. Gelation NPs (GN) were used to VEGF and PDGF delivery systems which exploited collagen and HA as NFs mesh. VEGF-loaded GN and bFGF HA solution and PDGF/VEGF-loaded GN and EGF collagen solution were electrospun during dual source dual power process. The obtained NPs-decorated NFs released four different GFs simultaneously (Fig. 4), which revealed the potential and capacity of NFs to deliver bioactive agents [101].

### Cell delivery and tissue engineering

Currently, one of the most effective methods for DM treatment is the replacement of IPCs using TE and regenerative medicine approaches. In this regard, IT, pancreatic TE, and cell replacement therapy by assisting fibrous scaffolds have emerged as powerful platforms with immense potential in DM treatment.

IT has been suggesting a perpetual treatment for DM patients which was providing some progress in clinical practice. Besides, It is divided into intrahepatic and extra-hepatic sites, proposing its advantages and disadvantages [104–107]. Several restrictions and limitations are present for the islet transplant procedure mostly maintaining viability and the functionality of islets after transplantation are limited by the loss of integrity and destruction of blood vessel networks as well as lack of proper access to nutrients and GFs [95, 108]. Moreover, the development and clinical application of IT have encountered some restrictions including insufficient donor source for transplantation, damage to the ECM of islets during the process of isolating by collagenase and patients need to take immunosuppressive agents for a lifetime [76, 109]. Therefore, it is essential to establish a suitable mechanically and biologically supportive scaffold/environment to improve islet culturing and transplantation efficiency. In this regard, NF systems open a new avenue to design advanced nanoenvironments for IT.

The preliminary study for developing NF scaffolds intended for islet cell transplantation was undertaken by Saahir Khan et al. [110]. They developed a glucagon-like peptide 1-mimetic peptide amphiphiles (PA) self-assembled NF gels to encapsulate RINm5f cells which could enhance insulin release and proliferation of encapsulated β-cells. Another PA NF, heparin mimetic nanofibrous gels, was employed in the long-term culture of islets as a new therapeutic approach for type 1 DM. The findings indicated that ECM-like environment by PA NFs provided with the ability to enhance islets viability, angiogenesis, and more efficient IT [95].

**Fig. 5:**
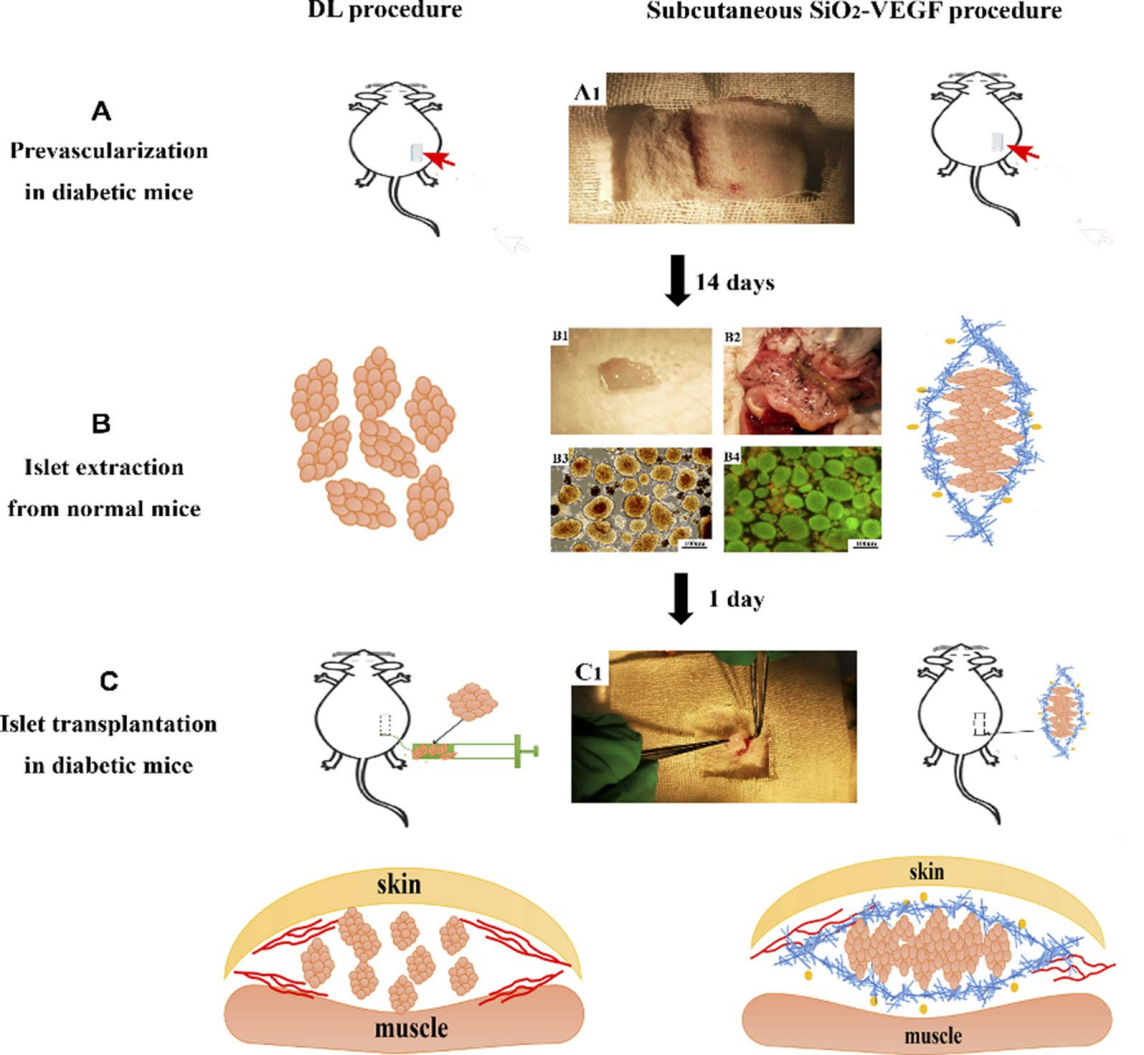
Schematic illustrating the device-less (DL) procedure and DL in the combination of SiO_2_-VEGF scaffolds for IT at subcutaneous. It comprised of 3 steps including: **A** A silicone/nylon catheter was pre-implanted subcutaneously (A1) (red arrow) to prevascularization and removed after 14 days; **B** islet isolation and SiO_2_-VEGF NFs wrapping (Blue mats = NFs, yellow dots = VEGF). (B1: wrapped islets; B2: expanded mouse islet for isolation; B3: syngeneic islets are isolated and collected (bar = 100 µm); B4. islet viability is detected by live/dead assay (bar = 100 µm)); **C** islet or wrapped islet (C1) transplantation in the prevascularized percutaneous cavity in diabetic mice. Reproduced with the permission from reference [113]

The possibility of PCL electrospun NF scaffold for an increment of growth and differentiation was confirmed as a good nanoenvironment for the differentiation of human-induced pluripotent stem cells (iPSCs) to endodermal cells (as precursors of hepatocytes and pancreatic cells) which revealed high viability, growth, and differentiation [70]. In another study, a biomimetic hybrid scaffold composed of electrospun SF and pancreatic decellularized ECM was developed for islet survival that had shown improved islet survival and promoted insulin secretion [111]. Whereas, the differentiation capability of conjunctiva mesenchymal stem cells (MSCs) into IPCs were studied on natural SF NFs and compared with synthetic PLA NF scaffolds which resulted in more pancreatic gene expression and higher insulin secretion by synthetic scaffolds [102]. Furthermore, the potential of unadulterated synthetic scaffolds including CA, PES, and polytetrafluoroethylene as active materials for islet cell encapsulation was evaluated. ES process can cause induced hydrophobicity to electrospun membranes which restrict cell attachment, preserving their inherent organization and cells maintained in an aggregated form compared to commercial ones [112]. In another pancreatic TE study, Yang B et al. subcutaneous space had chosen as an extra-hepatic site for IT. PVA/silicone NFs conjugated with VEGF were applied for subcutaneous IT [113]. The corresponding procedure is represented in Fig. 5. They found that modified NFs had no deleterious effect on cell viability, raised neovascularization, and induced mild inflammation, thereby the function of subcutaneously transplanted islets was augmented in diabetic mice.

Cells, scaffolds, and growth-stimulating factors are the main triad for TE and cell delivery. Numerous studies were undertaken to provide the right environment for pancreatic islet seeding and culture. At this point, NF scaffolds have attracted great attention for pancreatic TE due to their plentiful benefits. Sojoodi et al. reported the culture of rat islets on synthetic laminin-coated polyamide electrospun NFs that induced comparable gene expression to adult β cells and enhanced maintenance of functional islets [114]. Besides, coating β cell membranes onto PCL/poly-d-lysine electrospun NFs offered a natural environment recapitulating cell–cell interaction and significantly enhanced β cell function and proliferation rate [115]. In similar studies, the pancreatic differentiation capacity of induced iPSCs into IPCs on electrospun PES NFs [116] and collagen-coated PES NFs [117] were evaluated. The finding reveals that both scaffolds caused the expression of pancreatic tissue-specific markers and proteins at a high level and promoted differentiation of hiPSCs into IPCs.

In another study published by Enderami et al., PLA/PVA scaffolds were exploited as a substrate for the differentiation of iPSCs into IPC [118]. It was reported that the expression of pancreas-specific transcription factors considerably increased and IPCs formed spherical-shaped cell aggregations morphologically was similar to that of pancreatic islet cells [118]. Likewise, the same NF scaffolds were applied to promote the differentiation of adipose‐derived mesenchymal stem cells (ADMSC) into IPCs which resulted in a long time IPCs survival and function of cells [119]. The transplantation of pancreatic β cell precursors derived from human Wharton’s jelly MSCs by culturing on PLA/CS NF scaffold in a diabetic mice model resulted in a significant decline in blood glucose level and an increase in insulin levels after transplantation [120]. Moreover, the possibility of PLA/CS nano-scaffold treated with zinc oxide (ZnO) NPs to differentiate human endometrial stem cells (EnSC) into IPCs was assessed aiming at DM cell therapy [121].

As other NF scaffold, SF was constructed to simulate the pancreatic microenvironment for differentiation of iPSCs into IPCs which significantly evoked proliferation of the hiPSCs and its differentiation potential into IPCs [122]. Furthermore, IPC differentiation potency of hADSCs was examined on a hybrid NF scaffold composed of SF and PES polymers. This hybrid scaffold provided an in vivo-like 3D microenvironment, enabling to promote the proliferation and differentiation of hADSCs into IPCs [123]. Recently, a cell-co-polymer complex constructed from PHBV NFs was developed to differentiate human iPSCs into IPCs which increased the survival of iPSCs, the amount of IPC relevant genes and insulin secretion [124].

As a proper candidate for cell therapy and pancreatic TE in a diabetic's autologous transplantation, hADSCs, were applied to induce efficient differentiation into IPCs in the presence of PVA NFs and platelet-rich plasma. The differentiated IPCs showed the expression of beta cell markers of differentiation together with enhanced proliferation capability and insulin production [125]. Abazari et al. fabricated a 3D NF scaffold comprised of PCL and PVA polymers which provided a suitable synthetic ECM for the improvement differentiation of hiPSC to IPCs [126]. The differentiation potency of human endometrial stem cells (EnSC) from definitive endoderm cells on PAN scaffolds in culture medium containing Y-27632 molecules was also confirmed that the expression of pancreatic precursor markers elevated considerably. Subsequently, differentiated cells transplanted into the peritoneal cavity and/or injected via the tail of diabetic rats that the former caused a lower blood glucose concentration, and the latter was more effective in increasing the bodyweight of rats [127].

**Fig. 6:**
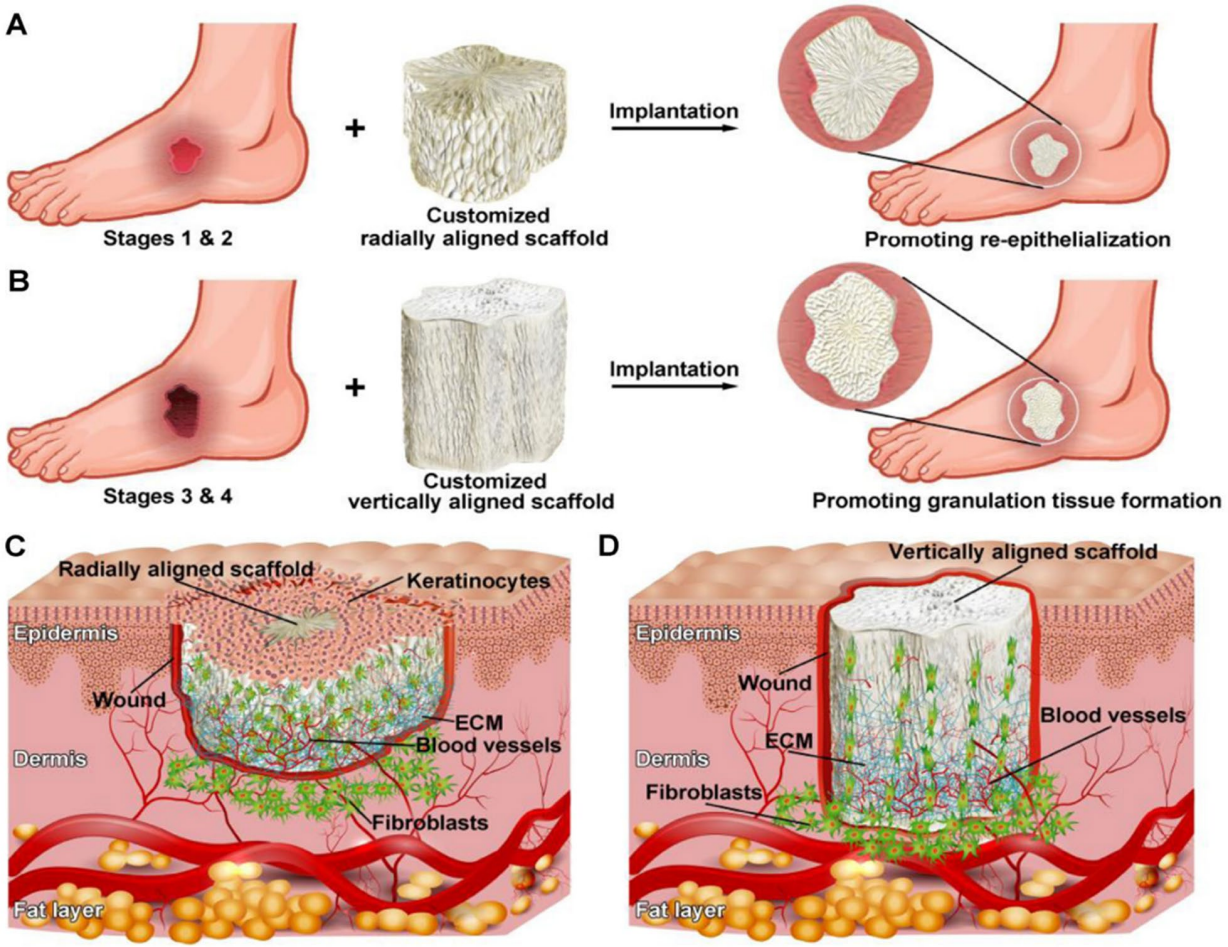
Schematic diagram describing the use of 3D scaffolds comprised of radially or vertically aligned NFs together with BMSCs for the treatment of DWs along with their potential mechanisms. **A** Illustration of radially aligned NFs applied for healing stage 0 and stage 1 DFU, with the potential mechanisms comprising improving angiogenesis, granulation tissue formation, ECM deposition, and re-epithelialization (**C**). **B** Illustration of vertically aligned NFs applied for healing stage 3 and stage 4 DFU, with the potential mechanisms comprising improving promoting granulation tissue formation, angiogenesis, and ECM deposition (**D**). Reproduced with the permission from Ref. [129]

Thanks to the ability to mimicking native ECM architecture using electrospun NFs, cell-based therapies have warranted enormous attention in DW healing and skin regeneration. PCL‐Gel scaffold associated to CD93^+^ hematopoietic stem cells was used as a suitable tissue‐engineered construct in DW healing. They found that the CD93^+^ cells are enabled to accelerate the healing and closing of diabetic ulcers by upregulating VEGF expression level and downregulating death‐associated protein kinase 1 expression level at the wound sites [128]. In a subsequent study, wound healing effects of 3D scaffolds comprised of radially-aligned and/or vertically-aligned NFs in conjunction with bone marrow MSCs were evaluated for DW healing applications [129]. 3D scaffolds were capable of enhancing the biological functions of laden cells, regulating the local inflammation, and allowed wounds to heal via promoting angiogenesis, improving the formation of granulation tissue, and increasing collagen deposition. Moreover, the results showed that radically-aligned scaffold could accelerate wound healing via the re-epithelialization of superficial wounds (DFU = stages 0–1) and vertically-aligned scaffold was able to enhance the formation of granulation tissues of deep wounds (DFU = stages 2–3) (Fig. 6) [129].

An exclusive multi-functional TE architecture was constructed with GO-PEG synthesized with quercetin (Que) and then loaded on the surface of artificial acellular dermal matrix (ADM) scaffolds (ADM-GO-PEG/Que), which can provide the biodegradable, biodegradable, cell-adhesive substrates with great stability. The resulting hybrid scaffold meaningfully promoted MSCs adhesion, proliferation, and differentiation into osteoblast and adipocyte as well as accelerated DW healing by promoting collagen synthesis and improving capillary construction [130].

Table 3 represents some of the main features of above-mentioned studies in this section. This table provides the characteristics of polymers/materials, incorporated/modified agents, and the fiber diameter of scaffolds as well as the differentiated cell type and their main achievement/applications.

**Table 3:** The characteristics of fibrous scaffolds applied in cell delivery and TE intended for treating DM

Type of polymer/material	Incorporated/modified agents	Diameter of fibers (nm)	Applied cell type to differentiation	Main achievement/application	Refs.
Glucagon-like peptide 1	–^a^	10	Rat insulinoma cells	A proper cell-encapsulating network for enhanced activity and proliferation of IPCs	[110]
Heparin mimetic peptide amphiphilic	VEGF and FGF2	20–30	Pancreatic islet	Nanofiber gel platform for islet culture and transplantation	[95]
PCL	–	200	hiPSCs	An ideal scaffold for differentiation of hiPSCs in 3D culture	[70]
SF and pig pancreatic decellularized ECM	–	97–707	Mouse islet	A promising candidate for pancreatic TE	[111]
CA, PES, and PTFE	–	365 ± 136 (CA), 224 ± 140 (PES), 261 ± 140 (PTFE)	–	Potential for islet cell encapsulation application	[112]
PVA /Silicone	VEGF	4–10	Mouse islet	The ECM to improve the vitality of subcutaneous islet transplantation	[113]
SF/PLA	–	–	Conjunctiva MSCs	A potential supportive matrix for islet TE	[102]
Polyamide	Laminin	–	Pancreatic β cell	Providing an ECM-like system for islet culture	[114]
PCL/poly-D-lysine	MIN6 cell membrane	50–280	Pancreatic β cell	As scaffolds to culture beta cells	[115]
PES	–	–	hiPSCs	A 3D matrix to enhance pancreatic differentiation of hiPSCs	[116]
PES	Collagen coating	–	hiPSCs	As a potential scaffold for pancreatic TE and regenerative medicine applications	[117]
PLLA/PVA	Oxygen modification	–	hiPSCs	As an ideal scaffold to provide a microenvironment for pancreatic differentiation	[118]
PLLA/PVA	–	–	hADSCs	A suitable option in pancreatic TE	[119]
PLA/CS	–	70,000	Human Wharton’s jelly MSCs	A precursor for cell transplantation for diabetes treatment	[120]
PLA/CS	–	70–100	EnCSs	An ideal scaffold for IPCs development for diabetes mellitus cell therapy	[121]
Silk	–	–	hiPSCs	A great potential to use in clinical pancreatic TE application	[122]
Silk/PES	–	–	hADSCs	As a supportive matrix to mimic 3D in vivo microenvironment	[123]
PHBV	–	900 ± 600	hiPSCs	As a promising cell-copolymer construct for pancreatic TE	[124]
PVA	Oxygen plasma	–	hADSCs	A new approach for pancreatic TE and β cell replacement therapies	[125]
PCL/PVA	–	–	hiPSCs	A new approach to beta-like cells replacement therapies and pancreatic TE	[126]
Polyacrylonitrile	–	250	Human endometrial cells	Transplantation of pancreatic precursor from endometrium for the treatment of diabetes	[127]
PCL/gelatin	–	–	Rat CD93^+^ hematopoietic stem cells	As a more appropriate tissue‐engineered construct in DW repair	[128]
PCL/pluronic-F-127	–	–	Bone marrow MSCs	Personalized 3D scaffolds with controlled structure for DW healing	[129]
Natural and artificial acellular dermal matrix	Graphene oxide- PEG-mediated quercetin	–	MSCs	A suitable architecture and environment for cell attachment and proliferation	[130]

^a^Not available data in the article

*PCL* polycaprolactone, *hiPSCs* human-induced pluripotent stem cells, *CS* Chitosan, *SF* silk fibroin, *VEGF* vascular endothelial growth factor, *FGF2* fibroblast growth factor 2, *TE* tissue engineering, *ECM* extracellular matrix, *CA* cellulose acetate, *PES* polyethersulfone, *PTFE* polytetrafluoroethylene, *PVA* polyvinyl alcohol, *PLA* polylactic acid, *PLLA* poly (l-lactic acid), *MSC* mesenchymal stem cell, *hADSC* human adipose-derived stem cells, *hESCs* human embryonic stem cells, *EnSCs* endometrial stem cells, *IPCs* insulin-producing cells, *PHBV* poly(3-hydroxybutyrate-*co*-3-hydroxyvalerate), *PEG* polyethylene glycol

### Therapeutics delivery for wound healing

Wound healing is one of the most sophisticated, highly regulated processes in the human body that is crucial for the restoration of tissue integrity and maintaining the barrier function of the skin. It involves the spatial and temporal synchronization and interplay of several cells, growth factors, and cytokines. It consists of sequential phases as the natural progression of a completely healed wound, including hemostasis, inflammation, proliferation, and remodeling, occurring within the immediate hours, days 1–4, days 4–21, and days 21-years after an injury, respectively [131]. Wounds can be caused by non-pathologic or pathologic conditions such as diabetes. A diabetic skin ulcer is one of the foremost complications of DM which can cause severe disorders e.g., DFU and even lower extremity amputations [132]. Numerous investigations were considered the potential procedures of therapeutic agents- loaded electrospun NF mats as a wound dressing to rapidly regenerate the structural and functional properties of injured skin in patients with DM [132–138]. Among these agents, antidiabetic drugs such as metformin (Met) and glibenclamide received much attention. Besides, the various synthetic and natural materials, including a blend of biocompatible and biodegradable polymers with or without the mentioned agents were extensively applied to fabricate NFs for wound healing applications.

For this purpose, we attempted to represent all capable polymers used as a wound dressing, including PVA/PCL, hydrogel-based, CS-based, cellulose-based, metal-based, and miscellaneous NF mats intended for DW. Besides, we investigated all in vitro and in vivo studies which examined the application of different therapeutic agents-loaded NF systems to treat DM or its complications e.g., DWs.

### PVA/PCL-based mats

#### PVA-based mats

Regarding small-molecule, Sena et al. produced PVA/PLA core–shell NFs containing Met that resulted in sustained release and good cell compatibility intended for DW healing [139]. Basha et al. prepared an electrospun fibrous scaffold containing PVA/curdlan blend by the addition of silver nitrate-based antimicrobial activity. The results from in vitro and in vivo studies presented noteworthy wound healing and better cell spreading and faster healing of removal wounds in diabetic rats, respectively [140]. In another study, Chouhan et al. investigated wound healing efficiency of dressings generated of various SF diversities blended with PVA intended for alloxan‐induced diabetic rabbit model. The results confirmed that the potential of non‐mulberry SF (NMSF)‐based bioactive dressings could regulate ECM deposition resulting in earlier and widespread treatment of chronic diabetic cutaneous wounds [141].

#### PCL-based mats

Curcumin (Cur), a plant-derived polyphenolic compound with anti-inflammatory, anti-bacterial, anti-oxidant, and angiogenic characteristics, was incorporated in two different NFs scaffolds, including PCL/GT and PCL, which enhance healing properties in both of them [142]. Fabrication of Cur-loaded PCL/gum tragacanth (GT) (PCL/GT/Cur) electrospun NFs with/without MSCs were investigated for wound healing in diabetic rats as well as antibacterial activity of these mats was studied. The resultant obtained from final mats with MSc approved that all quantification analysis of mats can be more potent than GT/PCL/Cur NFs [143]. In a similar study, the fabrication of electrospun PCL/Gel NFs loaded with Aloe Vera (AV) and Hypericum perforatum oil (HPO) was individually considered for the wound healing applications. Obtained results revealed that HPO-loaded mats played an effective role in healing DWs than AV [144]. In another study, Bixin, a carotenoid derived from the seeds of the Annatto plant (*Bixa orellana L*.) with antioxidant and anti-inflammatory activity, was loaded to PCL NFs as a wound dressing which alleviates scar tissues and accelerates DW healing [145].

Based on a novel approach, Zehra et al. designed to improve PCL-based oxygen-releasing electrospun mats and assess their value for enhancement of wound healing process in diabetic rats. The experimental results, including cell studies, chorioallantoic membrane, and histological assessment indicated that PCL-sodium percarbonate dressings could constantly generate oxygen within 10 days. The developed oxygen-generating mats could be applied for well-organized recovery of chronic DWs [146].

**Fig. 7:**
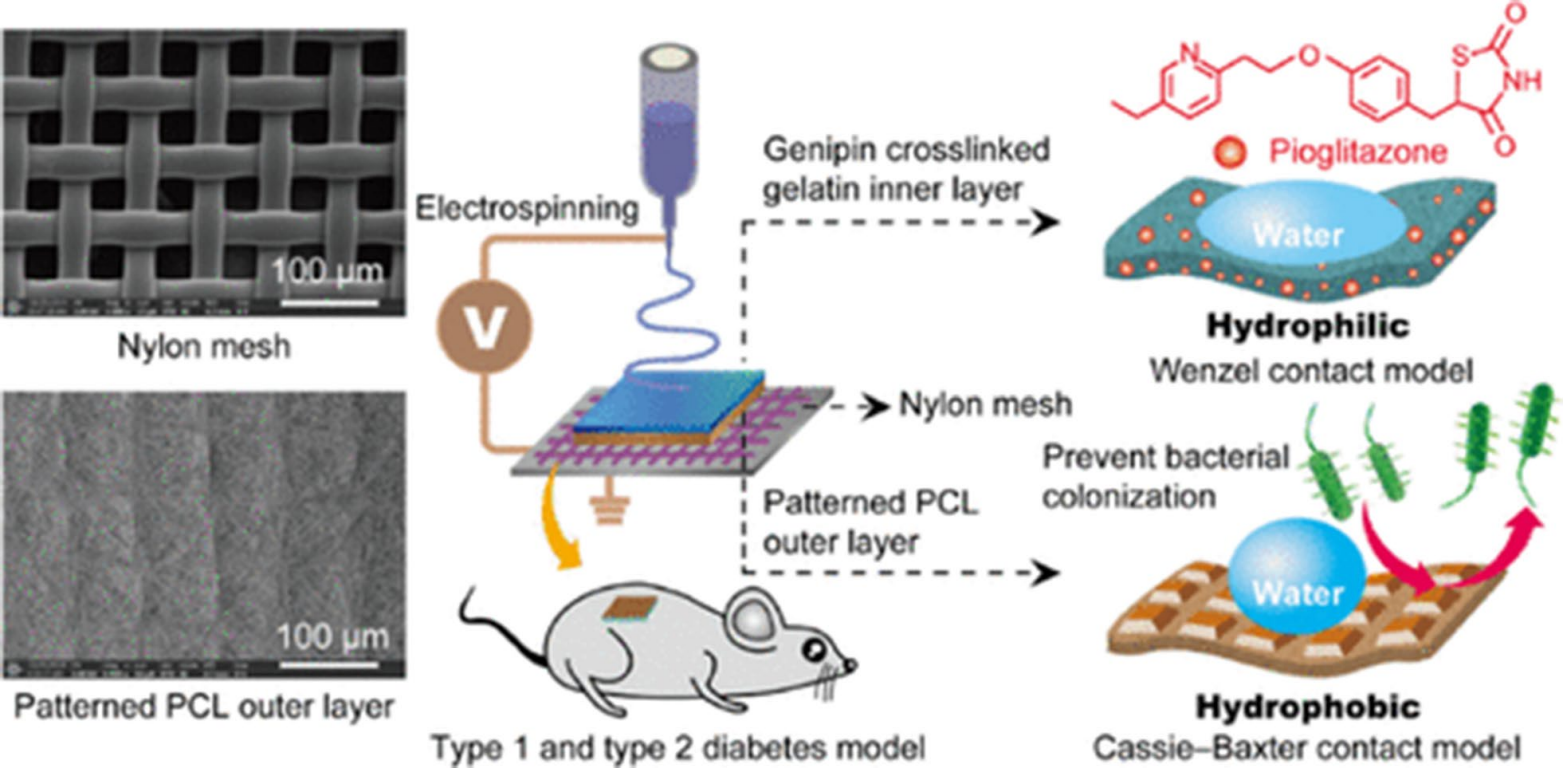
Conceptual design of an asymmetric wettable composite mat consisting PCL-Gel-Pio for DW healing. Reproduced with permission from Ref. [148]

Some studies have applied the combination of antidiabetic agents to enhance the wound healing properties of NF wound dressing. In a study conducted by Cam et al. pioglitazone (Pio), Met, and glibenclamide were loaded alone or in combination (Pio and Met or Pio and glibenclamide) with either CS/Gel/PCL or Polyvinylpyrrolidone (PVP)/PCL NF scaffolds by ES and pressurized gyration, respectively to compare their efficacy in DW healing. Their results suggested that CS/Gel/PCL scaffolds loaded by the combination of Pio and Met offer a suitable choice for DW dressing [147]. In a similar study, Yu et al. successfully prepared an asymmetric wettable composite mat with an extremely hydrophobic outer layer including PCL on nylon mesh with microporosity as well as the hydrophilic inner layer was attained using ES of Pio-merged Gel-Pio for DW healing (Fig. 7). The developed mat can boost the wound healing process by stimulating cell proliferation, angiogenesis, collagen deposition, and re-epithelialization [148].

Rehman Khan reported an electrospun poly (l-lactide-*co*-caprolactone) (PLCL) NF loaded with ZnO NPs and oregano essential oil, applying a novel loading approach, able to sustainedly co-deliver bioactive agents. The bioactive mats critically drove the angiogenesis through the expression of VEGF. Furthermore, the proposed system effectively completed the inflammatory cycle using inhibiting pro-inflammatory cytokines interleukin-6 (IL-6) and matrix metalloproteinases-9 (MMP-9) [149].

**Fig. 8:**
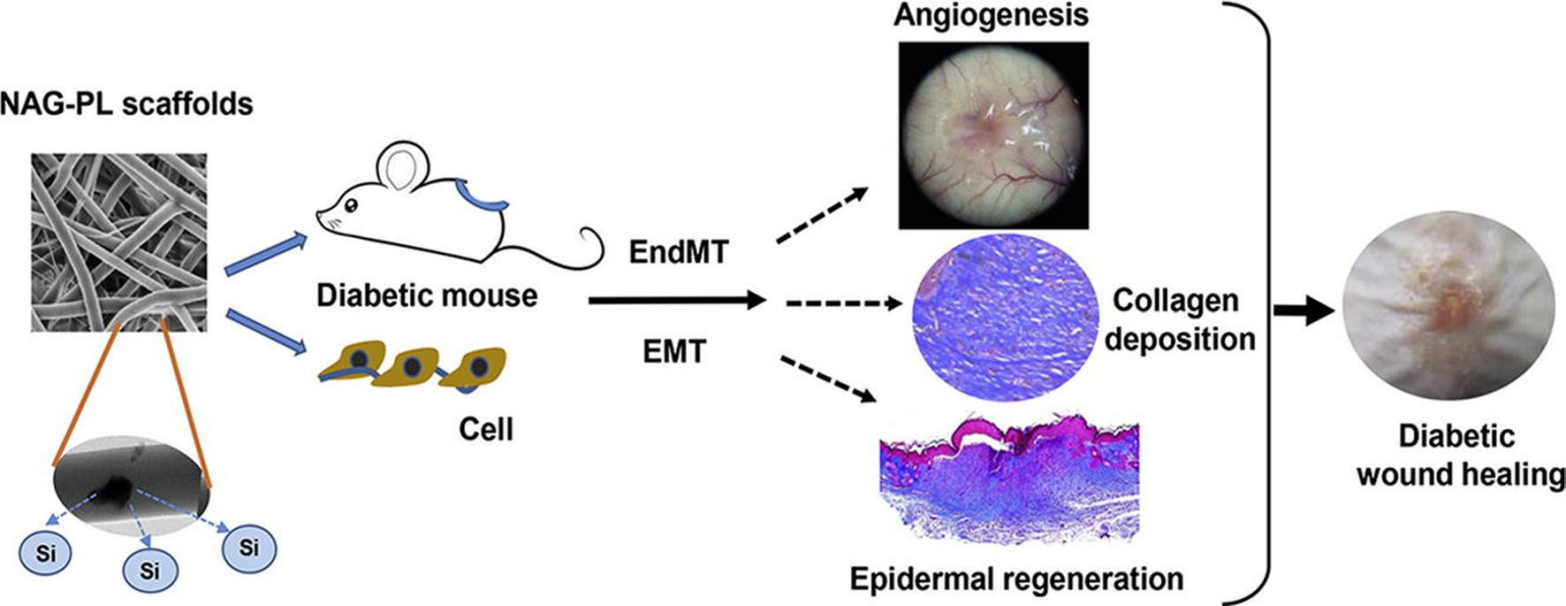
Conceptual design of Ca_7_P_2_Si_2_O_16_-loaded conducive PCL/Gel for wound healing process in diabetic mice. Reproduced with permission from Ref. [150]

Lv et al. designed a conducive PCL/Gel NF scaffold loaded by nagelschmidtite (Ca_7_P_2_Si_2_O_16_) particles, a silicate-based bioceramic, for DW dressing (Fig. 8). The composite scaffolds released the silicate ions in a sustained release manner during the degradation of NFs and showed accelerating wound healing by induction of collagen deposition, re-epithelialization, and angiogenesis [150].

Dimethyloxalylglycine (DMOG) can improve angiogenesis and tissue repair by inhibiting prolyl hydroxylases, an enzyme responsible for the degradation of hypoxia-inducible factor-1α (a key transcription factor which regulates angiogenesis in hypoxic conditions; e.g. wounds microenvironment). In one study, Goa et al. presented DMOG-loaded mono-axial and co-axial PCL/collagen for the wound healing process. The results confirmed that proposed system stabilized local hypoxia-inducible factor 1α levels in wounds and consequently enhanced the DW regeneration by speeding up re-epithelialization angiogenesis [151].

#### PVA-PCL mats

Fabrication and characterization of electrospun scaffolds including GT, PCL, and PVA were studied to heal diabetic ulcers. Histological analyses of mats holding stem cells into diabetic rats displayed tissue healing and regeneration consisting of re-epithelization and collagen formation within 15 days. Finally, the authors concluded that made-up NFs with remarkable mechanical and biological characteristics are promising scaffolds in wound healing of diabetic ulcers [152].

Gholipour-Kanani et al. fabricated different combinations of CS: PVA and PCL: CS: PVA electrospun biological scaffolds on diabetic dorsum skin wounds and diabetic foot wounds on rat models. Pathological results showed much better healing efficacy for test samples as well as proved the presence of more pronounced granulation tissues in scaffold-treated wounds compared with the control ones [153].

#### Nanofibrous hydrogel-based mats

**Fig. 9:**
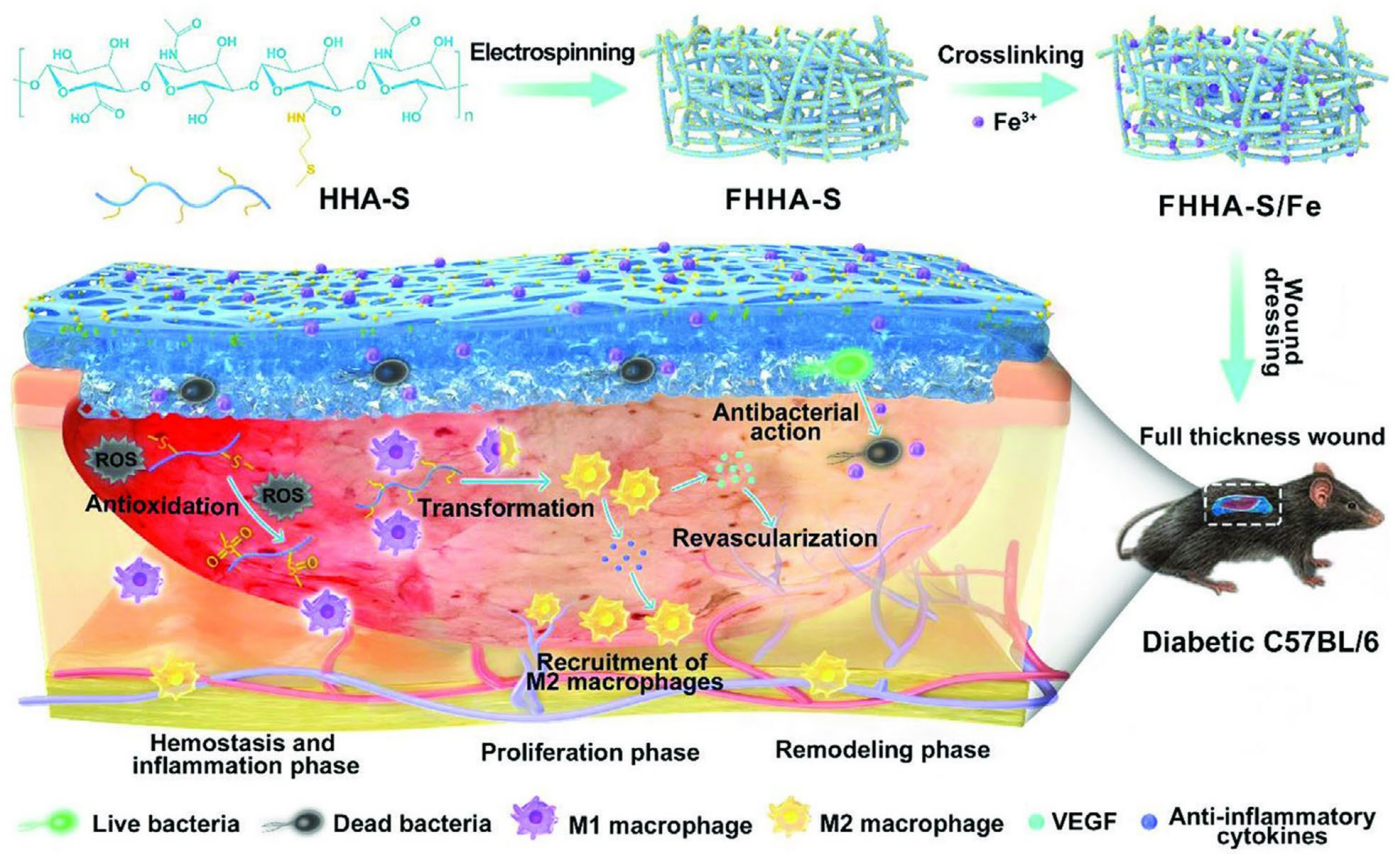
Schematic illustration of the absorbable thioether grafted hyaluronic acid nanofibrous hydrogel for synergistic modulation of the inflammation microenvironment to accelerate chronic DW healing. Illustration of the preparation procedure of FHHA-S/Fe, dressing of FHHA-S/Fe on full-thickness wound model in diabetic C57BL/6 mouse, and the mechanism of FHHA-S/Fe for enhanced chronic wound healing effect. Reproduced with permission from Ref. [133]

Liu et al. developed an absorbable NF hydrogel containing electrospun thioether grafted hyaluronic acid NFs (FHHA‐S/Fe) for a synergistic pattern of inflammation microenvironment to speed up chronic DW healing (Fig. 9). FHHA‐S/Fe treatment was more effective on the chronic DW model than that of FHHA/Fe without grafted thioethers, specifically in the initial wound healing stage. Hence, this simple dressing plan with fundamental dual modulation mechanisms of the wound inflammation microenvironment could play an impressive and safe therapeutic strategy for chronic DW [133].

In a similar study, self-assembling NF gel encapsulated-polydeoxyribonucleotide (PDRN) were fabricated to discover the treatment efficacy of chronic wounds in the diabetic animal model. The results obtained from human embryonic (HE) staining and immunohistochemical confirmed that poly-*N*-acetyl glucosamine (sNAG), and sNAG encapsulated-PDRN might ameliorate wound healing [154].

A novel wound care hydrogel-based product consisting of turmeric, oregano, and CS NPs diminishes inflammation, clear infection, and enhances wound healing in ulcers in diabetic rats. The proposed system can be applied as an effective scaffold in diabetic and non-DWs. This combination can also be applied as a potent new product that is antibacterial, anti-inflammatory, and antioxidant even though in low concentration [155].

TEMPO-oxidized sacchachitin nanofibers (SCNF) and microfludized SCNF were fabricated to form a 3D gel structure as an ideal hydrogel-based mat. The proposed hydrogel-based mats exhibited greater potentials in tissue regeneration as well as accelerated DW healing due to their exclusive physical and chemical properties [156]. Beta-glucan (βG), a major component of *saccharomyces cerevisiae* cell wall with immunomodulatory properties which can improve angiogenesis and tissue repair by inhibition of prolyl hydroxylases. In hydrogel-based study, βG-loaded hydroxypropyl methylcellulose and polyethylene oxide were prepared to improve DW healing [157].

#### Chitosan-based mats

Chogan et al. also showed that using a three-layer mat containing two PCL-CS layers on each side and an inside layer of PVA-Met could stimulate wound healing and mitigate skin fibrosis by down-regulation of genes involved in fibrosis [158]. Ahmadi Majd et al. fabricated PVA/CS electrospun NF wound dressings and used them to induce in diabetic rats. Obtained results revealed that PVA/CS NFs significantly improved wound healing in diabetic rats [159]. In another study, Ahmed et al. applied a mixture of CS, PVA, and ZnO as an effective possibility for an accelerated healing process owing to the wound healing activities of CS-PVA NFs and the antibacterial ability of ZnO [160].

Gel-based electrospun NFs, including Cur and Lithospermi radix extract were electrospun onto CS scaffolds to produce bilayer NF scaffolds as well as the final mat was applied to enhance the wound healing process in diabetic rats. The proposed mat indicated high anti-inflammatory effects and a satisfying recovery rate within 7 days for chronic DW [161].

**Fig. 10:**
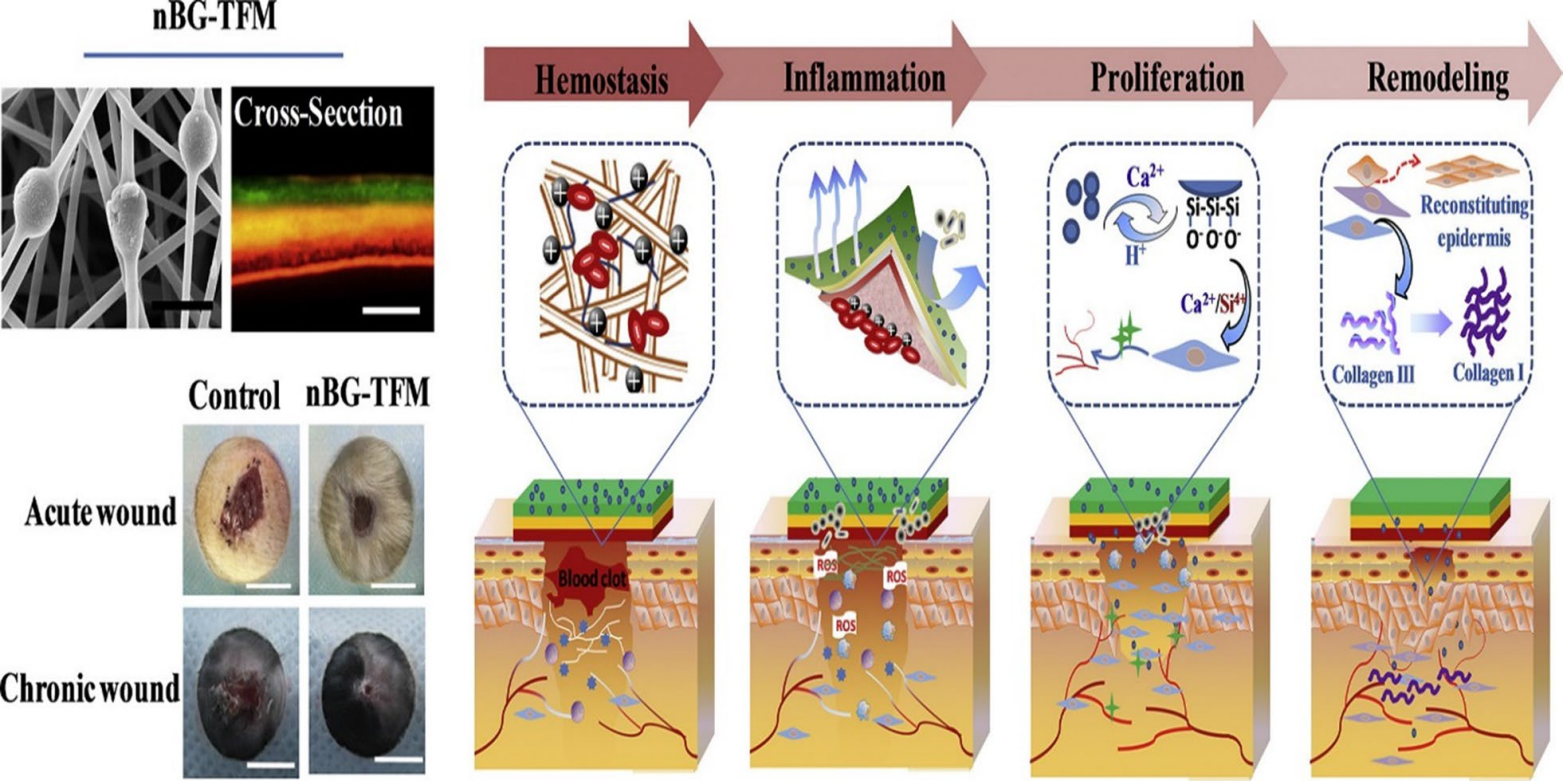
Schematic illustration of CS-PVA- electrospun NFs intended for chronic and acute wounds. Reproduced with permission from Ref. [162]

Chen et al. prepared nanobioglass integrated CS-PVA trilayer electrospun NF membrane (nBG-TFM). The as-prepared membrane indicated outstanding biocompatibility, antibacterial activity, and regeneration promotion effect (Fig. 10). The obtained system displayed shed new light on scheming functional wound dressings, which can ameliorate the healing of chronic wounds [162].

#### Cellulose-based mats

**Fig. 11:**
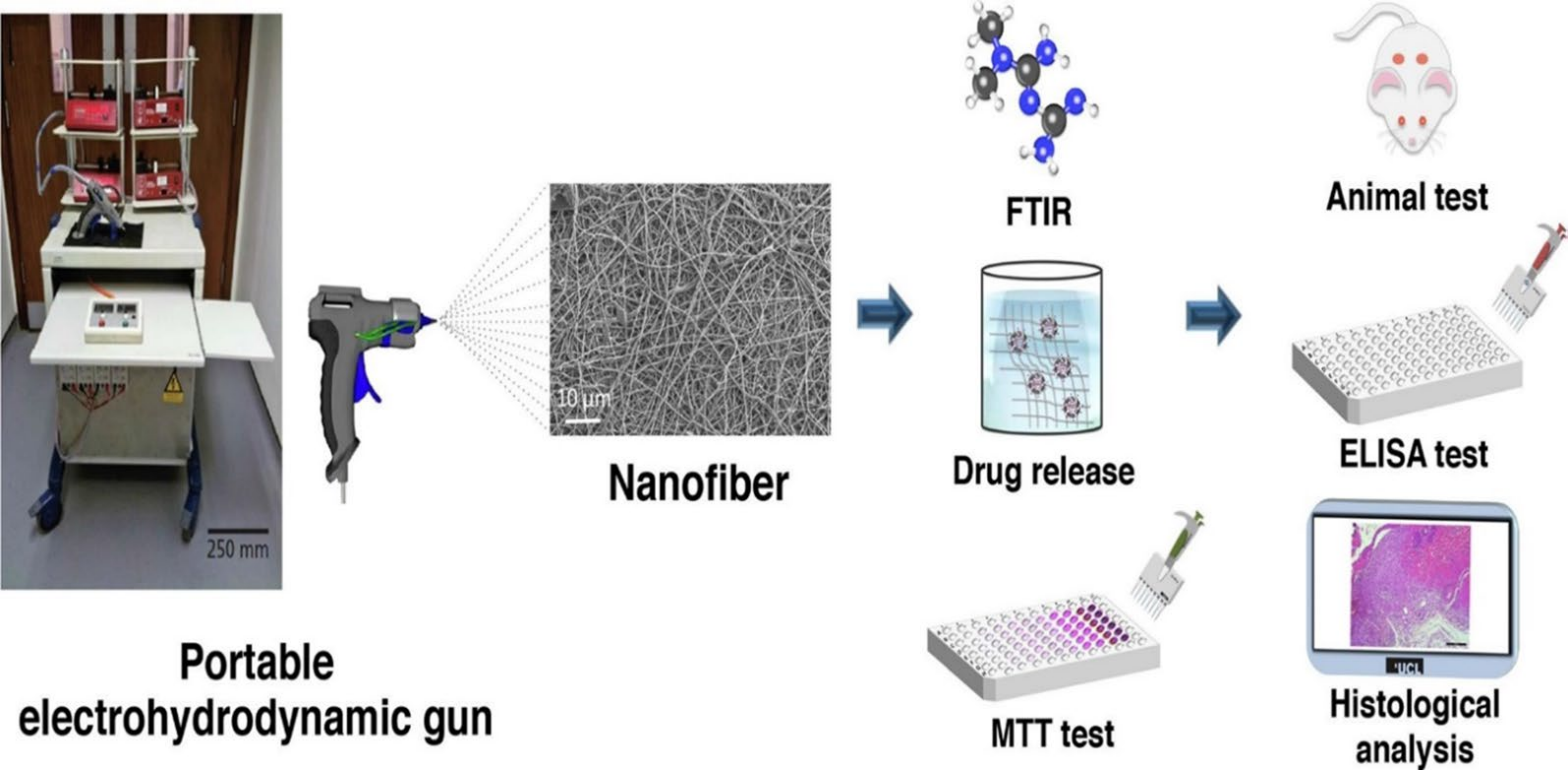
Schematic illustration of BC/Gel mats loaded with Gb and Met. Reproduced with permission from Ref. [163]

Bacterial cellulose (BC)/Gel NFs loaded with glybenclamide and Met were produced using a transportable electrohydrodynamic gun for DW healing by Emin Cam et al. (Fig. 11). This study’s results revealed both antidiabetic drugs-eluting dressing enhance DW healing. However, glibenclamide loaded scaffolds had better results [163].

Sesamol, one of the phenolic compounds of a sesame seed, was loaded into CA-zein composite NF that resulted in accelerated reepithelization and improvement in DW healing [164].

Almasian et al. prepared a new polyurethane (PU)-based NF scaffolds with different amounts of carboxymethyl cellulose (CMC) comprising *Malva sylvestris* extract, and they assessed their consequence on DW healing process. The extract-loaded PU/CMC presented high collagen deposition and neovascularization in treated DW compared with a gauze bandage and bare PU/CMC [165].

#### Metal-based mats

Li et al. prepared a cobalt-based metal–organic framework (MOF, ZIF-67) into micro-patterned PLLA/Gel NF scaffolds as a carrier for loading a small molecular drug (DMOG). The results confirmed that cobalt-based metal–organic framework as a dual cooperative controllable release system provides a new strategy for eliminating inflammation, enhancing collagen deposition and angiogenesis, and promoting DW healing [166].

**Fig. 12:**
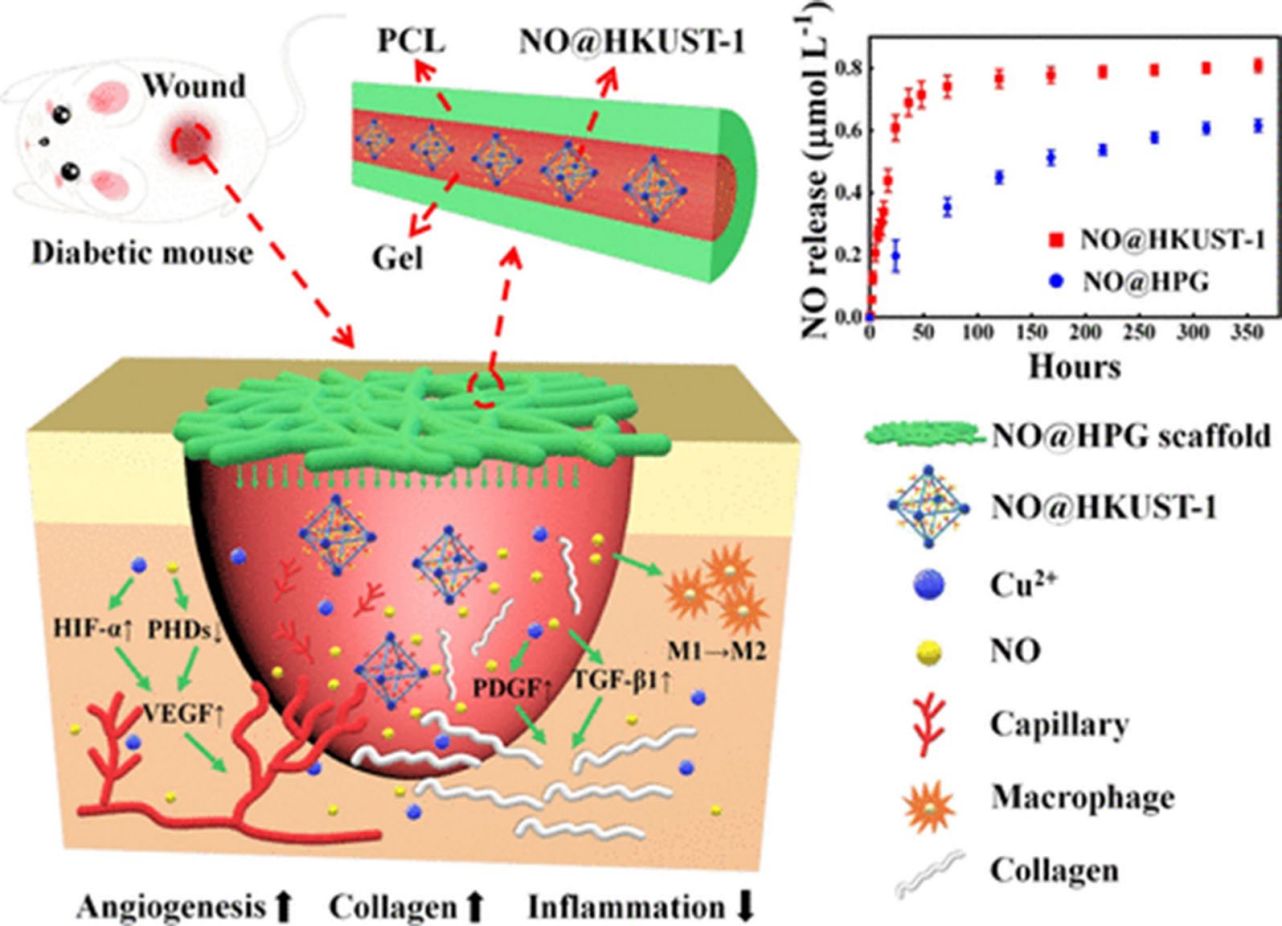
Schematic illustration of an electrospun copper-based MOF (HKUST-1) applied for the acceleration of DW healing process. Reproduced with permission from Ref. [168]

El-Lakany et al. used copper (Cu)-grafted GO-crosslinked zein scaffolds as a DW dressing and showed promising results [167]. In another study, an electrospun Cu-based MOF (HKUST-1) was presented as a NO-loading carrier, and a NO sustainable release system with the core–shell structure was considered (Fig. 12). The results confirmed that endothelial cell growth could meliorate and remarkably enhance angiogenesis, collagen deposition as well as anti-inflammatory property in the scaffolds which ultimately speed up DW healing [168].

Jiang et al. reported a spaced-oriented electrospun scaffold with silicon-doped amorphous calcium phosphate nanocoating on the surface (Si-ACP/PM). The study pointed out that Si-ACP/PM can notably improve the angiogenesis process for DW healing, as well as can display great potential for DW healing therapy [169].

#### PLGA-based mats

In some investigations, Met was incorporated in NF wound dressing to get advantages of their properties. In this regard, Met-eluting dressing made from PLGA by ES showed a controlled release profile over 3 weeks and also supported re-epithelialization and accelerated cutaneous wound closure in the early stages of DW healing [170, 171].

**Fig. 13:**
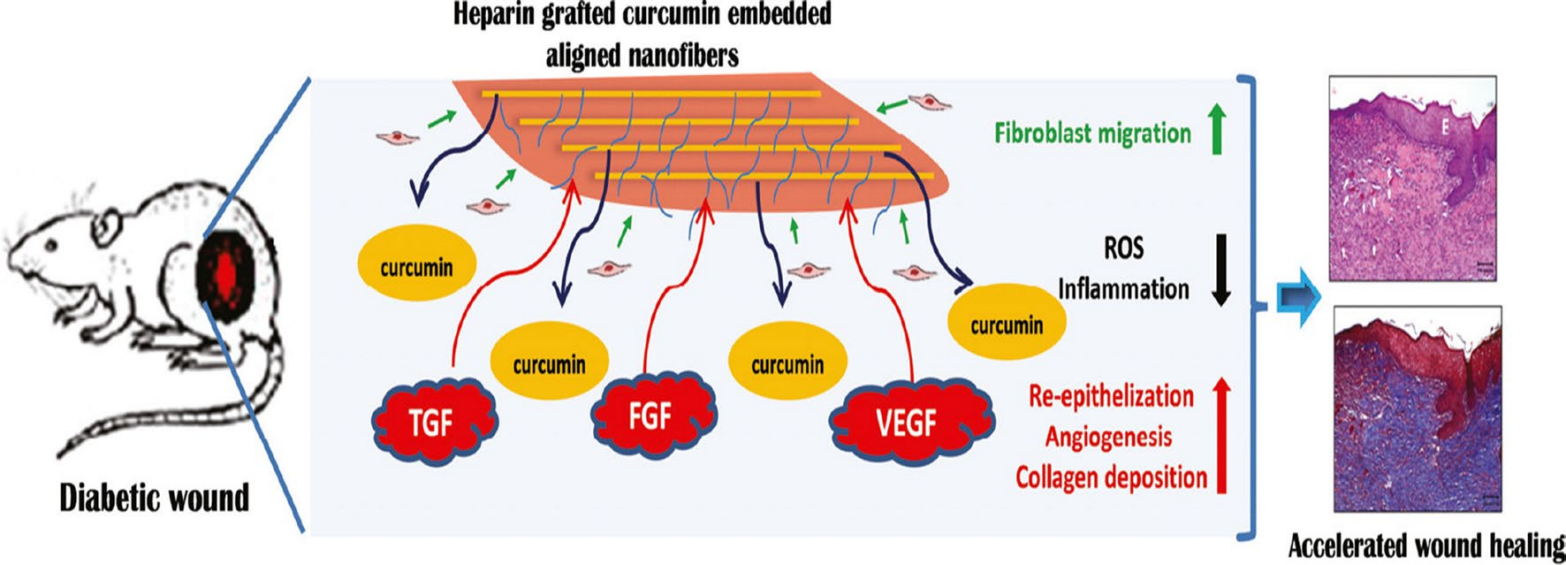
Conceptual design of heparin implanted Cur embedded aligned mats. Reproduced with permission from Ref. [172]

H.T. Liao et al. fabricated aligned Cur-loaded PLGA NF membranes (PC NFMs), followed by merging of heparin to produce PLGA/Cur (PCH) NFMs for DW healing (Fig. 13). Obtained results from this study confirmed that NFs along with grafted heparin and Cur could easily absorb the key GFs for the wound healing process, via lessening the high oxidative stress and the inflammatory cascade [172]. In a similar study, SF and PLGA were applied for the fabrication of a hybrid membrane. The results obtained from histopathological evaluation outcomes proved that these potent mats could potentially be applied for wound healing with or without biological agents [160]. In the case of large molecules, liraglutide (Lira), a glucagon-like peptide-1 (GLP-1) receptor agonist, was applied as an antidiabetic agent loaded on PLGA/Gel scaffold to accelerate DW healing. It was indicated that by applying Lira-loaded PLGA/Gel, meaningfully higher vascular density, higher collagen deposition level, and faster wound healing were achieved [173].

#### Miscellaneous-based mats

Pietramaggiori et al. prepared poly-*N*-acetyl glucosamine (pGlcNAc) fiber mats, and then they considered it for wound healing in the db/db mice. The obtained mat can be potentially applied as an effective agent for complex wounds owing to its blend of hemostatic and wound healing properties [174].

Kanji et al. proposed an aminated PES NF-extended human umbilical cord blood-derived CD34^+^ cells (henceforth CD34^+^ cells) real therapy, examined in cutaneous wounds for DW healing process in mice. They offered the proof of an innovative NF-expanded CD34^+^ stem cell healing for improving DWs by describing their cellular and molecular mechanisms [175].

In addition to antidiabetic agents, several other small molecules with varying biological characteristics were loaded into different NF scaffolds for wound dressing. In this regard, Han et al. showed that asiatic acid, an active ingredient of Centella asiatica (a Chinese medicinal herb), which have antioxidant, anti-inflammatory, and anti-bacterial properties when embedded in aligned porous PLLA electrospun fibrous scaffold could treat non-healing DWs [176].

**Fig. 14:**
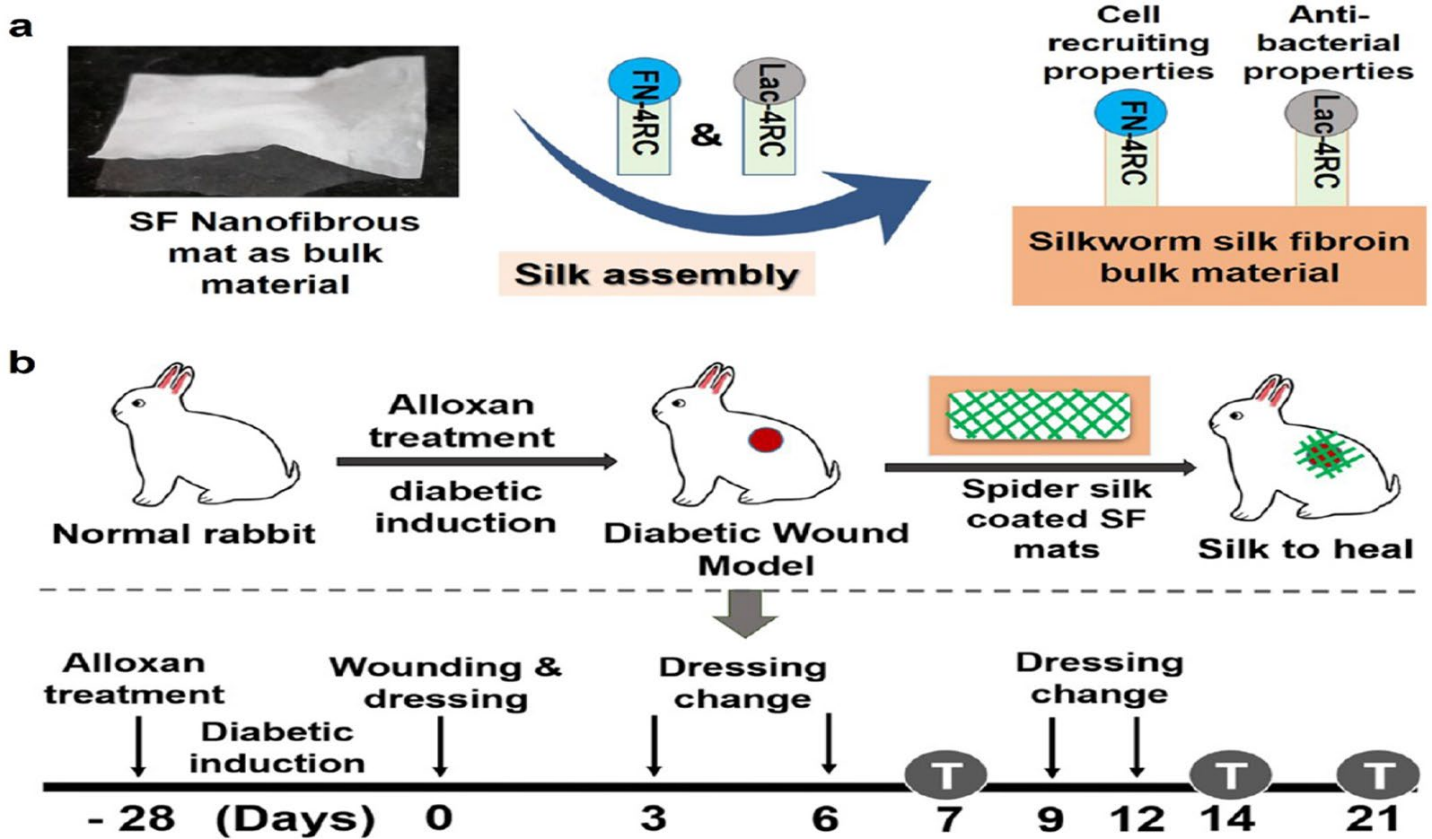
Schematic representation of the experimental design describing (**a**) methodology to prepare bioactive silk dressings by modifying spider SF proteins on top of SF nanofibrous mats and (**b**) approach of acting cutaneous wounds in a diabetic rabbit model by silk mats; DM condition was found for 28 days before wounding, mats were replaced after 3 days for 12 days, and groups were accomplished on day 7, 14, and 21 as indicated by (T) in the sketch. Reproduced with permission from Ref. [177]

SF derived from *Antheraea assama* silkworm (AaSF), coated with several recombinant spider silk fusion proteins over silk–silk connections, was employed as a bioactive NF mat for the wound healing process in diabetic rabbits (Fig. 14). The proposed system declared quick granulation tissue improvement, re-epithelialization, and well-organized matrix remodelling of wounds. Hence, the results obviously proved possible of achieved mats in earlier treatment of DWs [177]. In the case of diabetic rabbits, Elshazly et al. reported a novel formula of electrospun bioactive glass nanofibers (BGnf) containing B_2_O_3_, SiO_2_, and CaO for the improvement of oral mucosal wound regeneration. The findings indicated that obtained system can be applied as a sustainable oral cavity bioscaffold in a wet environment as well as can be applied for an immune-compromised disorder as DM [178].

Cui et al. also compared the effectiveness of topical doxycycline, an antibacterial agent, versus doxycycline-loaded PLA NF mats produced by ES technique for treatment/prevention of infection in DWs. Their results showed that doxycycline-PLA NF mats were superior to topical doxycycline to treat DWs, due to fewer side effects and better release profile [179].

Taking together, the results obtained from these studies revealed that proposed systems with different strategies are an ideal choice for scaffolds not only because of their biological characteristics but also owing to the possibility of using different therapeutic and preventive agents-loaded mats for DWs healing. Based on our observations, proposed mats were suggested as great tools with the ability to improve epidermal regeneration and re-epithelialization, promoting angiogenesis, and collagen deposition, and lessening inflammatory response. At last, it seems that antidiabetic agents and MSCs-loaded scaffolds can be a better choice for encapsulation in different mats so that we could apply for treatment of DWs, specifically chronic wounds.

Table 4 indicates several key features of above-mentioned studies. We tried to represent the most common characteristics, including the type of polymers/materials, the incorporated/modified agents, and diameter of NFs, applied cell type, and the main finding of proposed systems in this table.

**Table 4:** Findings of electrospun NF mats loaded with or without therapeutic agents for wound healing applications

Type of polymer/material	Incorporated/modified agents	Diameter of nanofiber (nm)	Applied cell type/animal	Main finding	Refs.
PVA/PLA	Met and FSP	621–681	HaCaT cell lines	Suitable properties for proliferation, and attachment of cells	[139]
Curdlan/PVA	1% AgNO_3_	92 ± 33	RAW 264.7 macrophage Cells/wister rats	Better cell viability, proliferation, and wound healing ability	[140]
SF/PVA	Non-mulberry SF, mulberry Bombyx mori SF	100–300	Endothelial cells/Alloxan induced diabetic rabbit	The functional benefit of regulating ECM secretion from fibroblast	[141]
PCL	Cur	200–1000	Epidermis-diabetic mice	Reducing inflammatory induction, as evidenced by low levels of interleukin‐6 release from mouse monocyte–macrophages seeded	[142]
PCL/GT	Cur	–^a^	Fibroblast, and epidermal cell/diabetic rats	Lessening blood glucose level	[143]
PCL/Gel	AV and HPO	–	TNF-α in serum samples/Wistar Albino male rats	Reducing oxidative stress index	[144]
PCL-based	Sodium percarbonate	473 ± 90	Thick epithelium-rats	Generation of chemical-based oxygen exposed angiogenesis stimulation	[146]
PCL/CS/Gel and PCL/PVP	Met and Pio	138.0 ± 42.5	Complete epidermis and dermis/type-1 diabetic rats	better regeneration and lower TNF-α and NF-κB levels than single drug therapies, good cytocompatibility with L929 cells, and an ideal area for the proliferation	[147]
PCL-based	Gel-Pio	144.9 ± 56.92	Epidermal cell-Type 1, and 2 diabetic mice	Promoting angiogenesis and cell proliferation and regulating the expression of MIP-2, VEGF, TNF-α, IL-1β, IL-6, MMP-9, and TGF-β	[148]
PLCL	ZnO NPs, and oregano essential oil	1040 ± 220	Epidermis, thick VEGF/stained tissue-rats	anti-inflammatory effect by down-regulating inflammatory-related gene expression	[149]
PCL/Gel	Nagelschmidtite (Ca_7_P_2_Si_2_O_16_) particles	800–2000	Epidermal cells-diabetic mice	Improving epidermal regeneration, angiogenesis, collagen deposition, and lessening inflammatory response	[150]
co-axial PCL /collagen	DMOG	391.42 ± 31.27	Epidermal and epidermis-T1DM rats	Enhancing the re-epithelialization, angiogenesis, and wound closure	[151]
PCL/GT/PVA	MSCs	130 ± 19	Epidermis/rats	Repair and regeneration including re-epithelization and collagen formation	[152]
PCL/PVA/CS	–	125 ± 12	Epidermis and dermis-rats	Higher rate of healing process	[153]
Absorbable nanofibrous hydrogel	FHHA‐S/Fe	60 ± 11	Epidermis/mice	Antioxidant properties and the capability of transforming the macrophage phenotype	[133]
Hydrogel-based	Gel encapsulated-polydeoxyribonucleotide	–	Human embryonic fibroblast cells, vascular endothelial cells/diabetic skin ulcer mouse model	levels of cytokines and angiogenic factors increased in the treatment groups	[154]
Hydrogel formulation	5% Turmeric, 1% Oregano, and 1% CS	211	Epidermis-rats	High anti-inflammatory, and antioxidants activities, as well as accelerated the healing process in pressure ulcers	[155]
TEMPO-oxidized SCNF and microfludized SCNF	Hydrogel-based mats including SCN5, T033SC, and T050SC	753.36 ± 103, 825.54 ± 109, and 496.54 ± 39	Endothelial cell marker, cluster of differentiation 31 (CD31)	Acceleration of the wound healing with forming nearly the same as normal tissue and providing the healed wound with a functional tissue	[156]
HPMC and PEO	βG	81 ± 39	db/db mice	βG-nanofiber significantly improved the healing as compared to the non βG-nanofibers	[157]
CS/PCL/PVA	MSc + Met	113 ± 43	Epidermis and dermis, and fibroblasts differentiate into myofibroblasts	Unique physico-chemical and biological properties of mats, introducing a slow-releasing and dual-functioning scaffold which reduces scar formation and accelerates the wound healing	[158]
CS/PVA	–	280	Epidermis and dermis-rats	Acceleration in diabetes wound healing	[159]
CS/PVA	ZnO	279.34 ± 7.23	Epidermis and scar tissue-rabbits	Useful dressing materials for DWs	[160]
Gel-based	Cur and Lithospermi radix extract	~ 100	STZ-induced diabetic rat mode	Enhancing collagen synthesis, TGF-β production, anti-inflammatory effect, and promoted the wound healing process	[161]
CS-PVA	Nano-bioglass	800 ± 400	Epidermis-rats	Upregulating growth factors of VEGF, TGF-β and downregulating inflammatory cytokines of TNF-α, IL-1β	[162]
Cellulose/Gel	Met and glybenclamide	220 ± 90, and 390 ± 10	Proliferation of L929 (mouse fibroblast) cells/T1DM rats	Observing the highest decrease of TNFα level	[163]
CA/zein	Sesamol	150–250	Diabetic mice	Reducing the expressions of inflammatory factors and IL-10, and sesamol, which can up-regulate IL-6 expression, promoting the growth and proliferation of keratinocytes	[164]
PU/CMC)	*Malva sylvestris* extract	277 ± 20	Epithelium/male Wister rats	Increasing macrophage infiltration, neovascularization activity, fibroblastic proliferation, and regeneration of collagenization and epithelium	[165]
Cobalt-based MOF ZIF-67-PLA/Gel	Dimethyloxalylglycine, DMOG	300–500	Epidermal cells and the complete epithelium-/STZ-induced diabetic mice	Enhancing angiogenesis, collagen deposition, elimination of inflammation in the DW, and promoting DW healing	[166]
Cu-GO	Zein	152.9 ± 14	Epidermal/diabetic rats	The highest transformation of granulation tissue, Epidermal reepithelialization	[167]
copper-based MOF, namely, HKUST-1	Nitric oxide as a gas medicine	~ 500	Complete epithelium/mice	Synergistically stimulate angiogenesis, promote collagen deposition, and inhibit inflammation	[168]
Si-ACP/PM	-	40	Human umbilical vein endothelial cells in vitro and epidermis and dermis-mice	Improved angiogenesis, reepithelialization, and collagen deposition in the wound site, which ultimately accelerates the progress of the DW healing	[169]
PLGA/collagen	Glucophage	203 ± 41	Epidermis-diabetic rats	Increasing collagen content and can act as an effective tissue-engineering scaffold for regenerating skin	[170]
PLGA	Met	443 ± 121	Epidermis-T1DM rats	Providing faster wound healing and better re-epithelialization	[171]
PLGA-based	Cur, and heparin	220 ± 16	Epithelium-rats	Acceleration of re-epithelization, higher angiogenesis, and collagen deposition	[172]
PLGA/SF	–	167 ± 50	Fibroblasts (L929) -rats	Decreasing the wound area in excision wound model in diabetic rats	[180]
PLGA/Gel	Lira	636 ± 198	Diabetic dermal wounds- rats	Promoting angiogenesis, AKT/GSK-3β/β-catenin pathways	[173]
pGlcNAc fiber mats	–	–	db/db mouse	Hemostatic and wound healing effects	[174]
Aminated PES	Human umbilical cord blood-derived CD34^+^ cells	–	Dermal and myofibroblasts-mice	Resolving inflammation, augmentation of angiogenesis, improving epithelialization and granulation tissue formation	[175]
Porous PLA	Asiatic acid	–	Diabetic mice	Accelerating re-epithelization, angiogenesis and ECM formation	[176]
GO-PEG	Quercetin as mediator and artificial acellular dermal matrix	402.71 ± 123.87	MSC/rats	Promotion of collagen deposition Enhancement of angiogenesis for DW healing at an early stage	[130]
*Antheraea assama* silkworm SF	Various recombinant spider silk fusion proteins	–	Complete epidermal-rabbits	Acceleration of the wound healing rate, improvement of angiogenesis, early re-epithelialization, and collagen synthesis	[177]
Bioactive glass nanofibres	–	500–900 nm	oral mucosal wound-T1DM-rabbits	Epithelial cell migration at a short time, providing a sterile wound bed and increasing VEGF precursor	[178]
PLA	DCH	424 ± 62	Epidermal and dermal layers-diabetic rats	enhancing the chronic wound healing, and have great superiority over topical coating of DCH solution	[179]

^a^Not available data in the article

*T1DM* type 1 diabetes, *PVA* poly vinyl alcohol, *PLA* polylactic acid, *Met* metformin, *FSP* fish sarcoplasmic protein, *SF *silk fibroin, *ECM* natural extracellular matrix, *PCL* polycaprolactone, *Cur* curcumin, *GT* gum tragacanth, *Gel* gelatin, *AV* Aloe Vera, *HPO* hypericum perforatum oil, *TNF-α* tumor necrosis factor alpha, *NF-κB* nuclear factor kappa B, *CS* chitosan, *PVP* polyvinylpyrrolidone, *Pio *pioglitazone, *PLCL* poly (l-lactide-co-caprolactone), *DMOG* dimethyloxalylglycine, *ZnO NPs* zinc oxide nanoparticles, *VEGF* vascular endothelial growth factor, *GT* gum tragacanth, *MSC* mesenchymal stem cells, *FHHA‐S/Fe* thioether grafted hyaluronic acid nanofibers, *TEMPO* 2,2,6,6-tetramethylpiperidinyloxy, *SCNF* sacchachitin nanofibers, *βG* beta-glucan, *HPMC* hydroxypropyl methylcellulose, *PEO* polyethylene oxide, *STZ* streptozotocin, *TNF-β* tumor necrosis factor-beta, *IL* Interleukin, *CA* cellulose acetate, *ECM* extracellular matrix, *PU* polyurethane, *CMC* carboxymethyl cellulose, *MOF* metal–organic framework, *DW* diabetic wound, *GO* graphene oxide, *Si-ACP/PM* silicon-doped amorphous calcium phosphate nanocoating on the surface, *PLGA* poly lactic-co-glycolide, *Lira* liraglutide, *pGlcNAc* poly-*N*-acetyl glucosamine, *PES* polyethersulfone, *PEG* polyethylene glycol, *DCH* doxycycline

### Electrospun NF mats and drug delivery systems

Despite numerous NFs in wound dressing applications, these biocompatible electrospun NFS have also been developed for drug delivery systems [168–170].In this regard, PVA NF patches loaded with linagliptin, an antidiabetic drug, were applied for sublingual administration [181]. In another example, the water solubility, drug release profile, and efficiency of repaglinide (an antidiabetic agent) for glycemic control were improved while loaded to PVA- PVP NFs [182]. Besides, Heydari-Majd et al. considered zein NFs as a delivery carrier for Barije (Ferula gummosa) essential oil (EO) which has antioxidant and antidiabetic (by inhibition of α-glucosidase and α-amylase enzymes) activity. Their results verified that, under the simulated gastrointestinal conditions, the release profile of Barije EO was found suitable for encapsulation using zein NFs and could consider as a novel tool for DM treatment [183]. In one study, Vildagliptin-eluting PLGA electrospun NFs was prepared as stents to treat diabetic vascular disease. The obtained membranes indicated great recovery of diabetic endothelial and reduction of smooth muscle cell (SMC) hyperplasia. Obtained results revealed that proposed stent could potentially accelerate the healing of diabetic arterial disorders [184].

It is concluded that the proposed systems in this section can be delivered in a controlled manner, and an adjustable glycemic control can be achieved via encapsulation of various antidiabetic agents in the scaffolds. Although for DM treatment, scaffolds loaded with therapeutic agents intended for oral administration could be more efficient than the topical ones, however, topical scaffolds were extensively applied due to simplicity and capability of sustained and controlled drug delivery to the wound site.

### Market size of advanced wound care

**Fig. 15:**
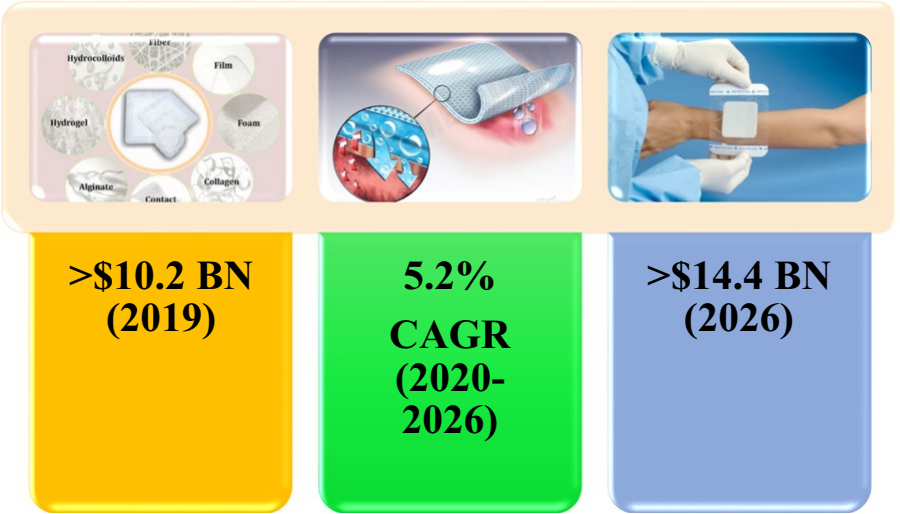
**T**he global advanced wound care market size from 2019 to 2026. *BN* billion, *CAGR* compound around growth rate

The global advanced wound care market size exceeded $10.2 billion (BN) in 2019 and is poised to raise at over 5.2% compound around growth rate (CAGR) between 2020 and 2026 (Fig. 15). The increasing prevalence of acute and chronic wounds in diabetes and obese population, as well as increasing geriatric population base that is at high risk of developing chronic wounds specifically DW, will augment the market size [185].

The strategic trends in the market are the growing consumption of NF-based wound dressing and rising demand for incorporation dressings. Incorporation dressings including natural and chemical agents, such as silver alginate, collagen hydrochloride, and silver collagen, averts infection and speeds up wound healing process. Thanks to the great preventive and therapeutic efficiency of advanced wound dressings, it is a foremost trend in the advanced wound care market.

### Limitations, future perspectives, and conclusions

Even though various studies showed that electrospun NF mats play a key role in wound healing applications, ES process possesses definite limitations in elastic possibility due to its conservative setup that is typically fairly bulky and extremely dependent on a plug. The selection of suitable blend polymers and therapeutic agents has still remained the main challenge for wound dressing fabrication. Furthermore, some restrictions and limitations for islet transplant procedure are restricted by the loss of integrity and demolition of blood vessel networks as well as insufficient access to nutrients and GFs.

The wound healing process using dressings is growing faster progressively owing to an increase in the world population ($45.5 billion by 2024). Hence, chronic wound cases also need to improve proper medication, which can effectively conquest the gentle wound healing process and hinder wound infection. Consequently, physicians, pharmacologists, biomedical engineers, and other relevant fields should research together in this path for better releasing of the wound healing process, enhanced drug efficacy, and enhanced drug delivery systems. This research review article defines the wound healing process using various nanosystems, including electrospun NFs for DWs healing. These systems, including therapeutic and preventive agents play a pivotal role to protect and improve the wound healing process specially DWs. Besides, emerging smart mats can also promote DWs healing and real-time monitoring. The assessment of the wound healing process indicates that electrospun NF mats provide better features compared to common mats in respect of cost, healing time process, and effective and sustainable drug delivery. In addition, NF-based systems typically act as non-invasive, biodegradable, biocompatible systems without notable side effects, which have been more considered in wound healing promotion while compared to the other systems. It is also believed that obtained mats can pave the route of preclinical and clinical studies in TE and regenerative medicine, exclusively wound healing process.

Blending various polymers using more effective cross-linking methods to produce enhanced scaffolds that support an optimal wound healing process was developed in recent years. As alluded to above, natural polymers, including cellulose and its derivatives, CS, hyaluronic acid, collagen, SF, and synthetic polymers including PVP, PVA, PLGA, PLA, PCL, PEG, PVP, PU were merged together with or without cross-linkers to apply for wound dressing applications. Besides, therapeutic wound dressings were inspected to powerfully deliver therapeutic and preventive agents that had earlier been revealed to promote the wound healing process, specifically DWs. A clear approach for the reduction of infection is applying electrospun NFs loaded with antidiabetics/antimicrobial compounds, platelet-derived ingredients, MSCs, GFs, and peptides to control up-regulation of GFs and ECM secretion from fibroblast, and down-regulation of inflammatory cytokines and inflammatory-related gene expression in DWs to accelerate the healing process. Moreover, re-epithelialization, angiogenesis, wound closure, epidermal regeneration, and collagen formation have been promoted in most cases. Regarding the encapsulation of natural extracts, several studies displayed notable potential in the considerable healing of DWs; however, these outcomes do not recommend a fruitful choice since the efficiency of herbal extract, adjustment of their impact has remained as key challenges. Hence, relevant research studies will definitely focus on developing more potent and less costly biocompatible and biodegradable therapeutic mats that provide great healing to DWs to promote patient treatment and quality of life. We hope that therapeutic and preventive electrospun NF mats have opened a door for exploring novel wound healing processes to be applied in DWs, as well as many other expectations.

## Data Availability

Not Applicable.

## Abbreviations

3D: Three dimensional
ADMSC: Adipose‐derived mesenchymal stem cell
AV: Aloe vera
AaSF: Antheraea assama silkworm
bFGF: Basic fibroblast growth factor
βG: Beta-glucan
BC: Bacterial cellulose
BGnf: Bioactive glass NFs
CA: Cellulose acetate
CS: Chitosan
CTGF: Connective tissue growth factor
CNC: Cellulose nanocrystal
Cur: Curcumin
CMC: Carboxymethyl cellulose
DM: Diabetes mellitus
DW: Diabetic wound
DMOG: Dimethyloxalylglycine
PDRN: Polydeoxyribonucleotide
ECM: Extracellular matrix
ES: Electrospinning
EGF: Endothelial growth factor
EnSC: Endometrial stem cell
NF: Nanofiber
FDA: Food and Drug Administration
FGF2: Fibroblast growth factor 2
FHHA‐S/Fe: Thioether grafted hyaluronic acid nanofibers
FSP: Fish sarcoplasmic protein
GF: Growth factors
Gel: Gelatin
GO: Graphene oxide
GN: Gelation nanoparticles
GT: Gum tragacanth
HA: Hyaluronic acid
HBPA: Heparin-binding peptide amphiphile
hiPSCs: Human-induced pluripotent stem cells
HPO: Hypericum perforatum oil
HE: Human embryonic
IPCs: Insulin producing cells
IT: Islet transplantation
iPSCs: Induced pluripotent stem cell
LPEI: Linear polyethyleneimine
Lira: Liraglutide
MSC: Mesenchymal stem cells
Met: Metformin
MOF: Metal–organic framework
NP: Nanoparticle
nBG-TFM: Nanobioglass integrated CS-PVA trilayer electrospun NF membrane
PCL: Polycaprolactone
PM: PuraMatrix™
PM-Isol: PM-insulin sol
PVA: Poly vinyl alcohol
PLA: Polylactic acid
PLGA: Poly lactic-*co*-glycolide
PEG: Polyethylene glycol
PES: Polyethersulfone
PHBV: Poly 3-hydroxybutyrate-co-3-hydroxyvalerate
PAN: Polyacrylonitrile
phEGF: Plasmid human epidermal growth factor
PELA: Poly(ethylene glycol)-poly(dl-lactide)
PDGF: Platelet-derived growth factor
PA: Peptide amphiphiles
PHBV: Poly 3-hydroxybutyrate-*co*-3-hydroxyvalerate
PDRN: Polydeoxyribonucleotide
PEO: Poly (ethylene oxide)
PU: Polyurethane
pGlcNAc: Poly-*N*-acetyl glucosamine
PVP: Polyvinylpyrrolidone
Pio: Pioglitazone
PLCL: Poly (l-lactide-*co*-caprolactone)
SF: Silk fibroin
SCNF: Sacchachitin nanofibers
Si-ACP/PM: Silicon-doped amorphous calcium phosphate nanocoating
sNAG: Surface poly-N-acetyl glucosamine
TE: Tissue engineering
VEGF: Vascular endothelial growth factor
ZnO: Zinc oxide
